# Controlling the Antimicrobial Action of Surface Modified Magnesium Hydroxide Nanoparticles

**DOI:** 10.3390/biomimetics4020041

**Published:** 2019-06-25

**Authors:** Ahmed F. Halbus, Tommy S. Horozov, Vesselin N. Paunov

**Affiliations:** 1 Department of Chemistry and Biochemistry, University of Hull, Hull HU67RX, UK; a.f.al-mamoori@2014.hull.ac.uk (A.F.H.); t.s.horozov@hull.ac.uk (T.S.H.); 2 Department of Chemistry, College of Science, University of Babylon, Hilla, Iraq

**Keywords:** Mg(OH)_2_NPs, magnesium hydroxide, polyelectrolytes, poly (styrene sulfonate), poly (allyl amine) hydrochloride, antimicrobial nanoparticles, algae, yeast, bacteria, *C. reinhardtii*, *S.cerevisiae*, *E. coli*

## Abstract

Magnesium hydroxide nanoparticles (Mg(OH)_2_NPs) have recently attracted significant attention due to their wide applications as environmentally friendly antimicrobial nanomaterials, with potentially low toxicity and low fabrication cost. Here, we describe the synthesis and characterisation of a range of surface modified Mg(OH)_2_NPs, including particle size distribution, crystallite size, zeta potential, isoelectric point, X-ray diffraction (XRD), dynamic light scattering (DLS), scanning electron microscopy (SEM), thermogravimetric analysis (TGA), energy dispersive X-ray analysis (EDX), Fourier transform infrared spectroscopy (FTIR), and transmission electron microscopy (TEM). We explored the antimicrobial activity of the modified Mg(OH)_2_NPs on the microalgae (*C. reinhardtii*), yeast (*S. cerevisiae*) and *Escherichia coli (E. coli*). The viability of these cells was evaluated for various concentrations and exposure times with Mg(OH)_2_NPs. It was discovered that the antimicrobial activity of the uncoated Mg(OH)_2_NPs on the viability of *C. reinhardtii* occurred at considerably lower particle concentrations than for *S. cerevisiae* and *E. coli*. Our results indicate that the antimicrobial activity of polyelectrolyte-coated Mg(OH)_2_NPs alternates with their surface charge. The anionic nanoparticles (Mg(OH)_2_NPs/PSS) have much lower antibacterial activity than the cationic ones (Mg(OH)_2_NPs/PSS/PAH and uncoated Mg(OH)_2_NPs). These findings could be explained by the lower adhesion of the Mg(OH)_2_NPs/PSS to the cell wall, because of electrostatic repulsion and the enhanced particle-cell adhesion due to electrostatic attraction in the case of cationic Mg(OH)_2_NPs. The results can be potentially applied to control the cytotoxicity and the antimicrobial activity of other inorganic nanoparticles.

## 1. Introduction

The increased proliferation of infectious illnesses that are caused by microorganisms found in food packaging, medical devices, water treatment systems, and domestic appliances has elicited increased interest [1,2,3]. The increased resistance of microorganisms against current biocides has caused great concern, particularly for individuals of compromised immune systems [4,5]. This has prompted expanded efforts to investigate new types of nanomaterials as antibacterial agents [6,7,8], which do not rely on the existing pathways of antimicrobial resistance. Recent studies have been concentrated on antibacterial inorganic nanoparticles, for example, metal oxide nanoparticles, like ZnO, MgO, CuO, Cu_2_O, Al_2_O_3_, TiO_2_, CeO_2_, and Y_2_O_3_; metals, e.g., copper, silver, gold etc., metal hydroxides, such as Mg(OH)_2_ as well as colloids made from biodegradable materials, such as chitosan, lignin, and dextran, loaded with antibacterial agents [9]. Mg(OH)_2_NPs have been successfully deployed as antifungal and antibacterial agents towards different microorganisms [10,11,12] and there are indications that they can be highly effective [13]. Mg(OH)_2_NPs have attracted significant attention over the years due to their wide applications in different fields as an environmentally friendly material with low cost of production [14,15,16,17] and they may be potentially used in pharmaceutical formulations [18,19,20,21]. However, a limited number of studies have investigated the antimicrobial effects of Mg(OH)_2_NPs and reported that the in vivo toxicity values are low, thus demonstrating that it has a non-toxic effect on humans in sensible amounts [22]. Recently, it has been reported that Mg(OH)_2_NPs were effective as antibacterial agents towards several bacteria, including *E. coli*, *S. aureus*, *P. aeruginosa,* and *B. phytofirmans* [23,24,25,26,27,28], and a number of studies have been focused on this new and effective antimicrobial agent [17]. Dong et al. have investigated the antibacterial action of Mg(OH)_2_NPs on *Burkholderia phytofirmans* and *Escherichia coli* [24]. Their results indicated that the Mg(OH)_2_NPs suspensions are effective towards *B. phytofirmans* and *E. coli.* Their study has also examined the role of the OH^-^ and Mg^2+^ ions, which are naturally present in the Mg(OH)_2_NPs suspension, on the antimicrobial action. They showed that an alkaline medium of pH 10.4, as well as an equivalent amount of Mg^2+^ ions in the aqueous solution. could not kill the bacteria [24]. They have also indicated that Mg(OH)_2_NPs can kill *E. coli,* even in dark conditions, which suggested that no photocatalytic properties are involved in their antibacterial action [23]. Hence, the antibacterial mechanism of Mg(OH)_2_NPs seems to be very different to those of other metal and metal-based compounds [28,29,30,31]. Pan et al. synthesised Mg(OH)_2_NPs from three different precursors (MgCl_2_, MgSO_4_, and MgO) and tested their antibacterial efficiency towards *E. coli* as a model Gram-negative bacteria [17]. Bactericidal examinations indicated that the antibacterial activity of Mg(OH)_2_NPs was inversely related to particle size. Their results also revealed that the ability of Mg(OH)_2_NPs to adhere on the bacterial cell walls decreased in the order: Mg(OH)_2___MgCl__₂_ > Mg(OH)_2___MgSO__₄_ > Mg(OH)_2___MgO_, which showed that the toxicity of the produced Mg(OH)_2_NPs may be caused by the electrostatic interaction induced by secondary adsorption of counter-ions. This means that the type of precursor magnesium salt that is used to produce the Mg(OH)_2_NPs by hydrolysis can greatly influence their antimicrobial properties by the secondary absorption of counter-ions on the particles surface. These authors propose that Mg(OH)_2_NPs adsorb on the negatively charged bacterial cell wall and somehow disrupt its integrity, and increase its permeability, which kills the bacteria [17]. 

**Figure 1:**
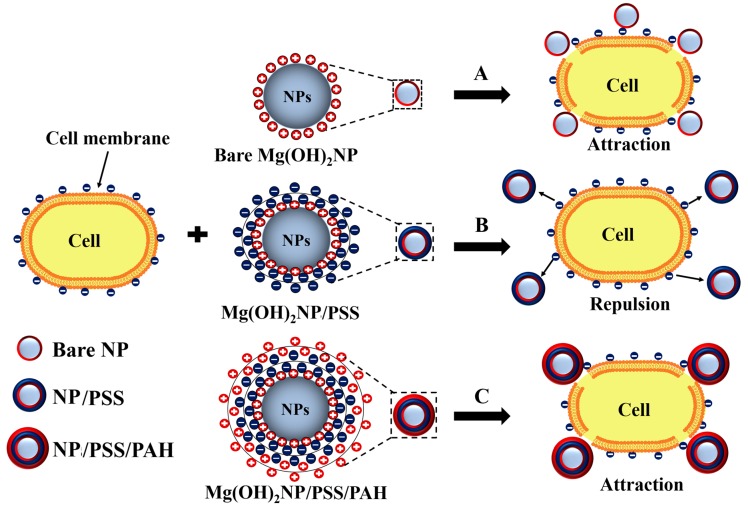
Schematic diagram showing the various contacting patterns between the bare and polyelectrolyte-coated Mg(OH)_2_NPs on cells. (**A** and **C**) The adhesion of the uncoated and cationic polyelectrolyte-coated Mg(OH)_2_NPs to the cell wall surfaces is favoured due to their opposite surface charges. (**B**) The interaction between the anionic outer surface of the cell membrane and the Mg(OH)_2_NPs coated with anionic polyelectrolyte is repulsive. The cationic Mg(OH)_2_NPs and Mg(OH)_2_NPs/PSS/PAH nanoparticles are expected to be more effective against the microbial cells than the anionic Mg(OH)_2_NPs/PSS particles.

In this article, we investigate the role of the polymer coating in the antimicrobial activity of Mg(OH)_2_ nanoparticles that were synthesised by the direct precipitation method. Three different types of microorganisms, *C. reinhardtii*, *S.cerevisiae* cells, and Gram-negative *E. coli* were used to examine the antimicrobial activity of the surface modified Mg(OH)_2_NPs. In this study, we are interested in using the surface functionalized Mg(OH)_2_NPs as innovative anti-algal, anti-bacterial, and antifungal agents. As *C. reinhardtii* is a typical representative of the algae genre and *S. cerevisiae* is a fungal microorganism, they are a good proxy for these assessments. We explored the relationship between the antifungal and antibacterial effect of the particle size, surface charge, in addition to their adhesion to the microbial cell wall. The size of the Mg(OH)_2_NPs is likewise essential for their potential activity, as smaller particles have higher portability to relocate between the biological compartments [32]. Moreover, the surface charge of the Mg(OH)_2_NPs determines their ability to electrostatically interact with the biological membranes. The present study investigates the impact of the Mg(OH)_2_NPs concentration, their zeta potential, and particle size on the viability of *C. reinhardtii, S.cerevisiae,* and *E. coli* at different exposure times. We explore the antimicrobial activity and the nanoparticle internalisation between *C. reinhardtii, S.cerevisiae,* and *E. coli*. In our experiments with surface functionalized Mg(OH)_2_NPs on microbial cells, which were systematically done on *C. reinhardtii*, *S.cerevisiae,* and *E. coli*, we have tested their effect in the absence of growth media whose components may potentially interfere with the particle surface charge. This would lead to ambiguity in the results, depending of the media composition and concentration. To avoid this, we remove the microbial cells from the media prior to testing the effect of the surface functionalized Mg(OH)_2_NPs on them. We investigate the antibacterial activity of Mg(OH)_2_NPs that are coated with anionic and cationic polyelectrolytes. Our working hypothesis is that coating the Mg(OH)_2_NPs with cationic polyelectrolytes may enhance their antimicrobial activity, while coating them with anionic polyelectrolytes as an outer layer may lead to decreased antibacterial activity because of their electrostatic repulsion from the bacterial cell wall (Figure 1).

## 2. Materials and Methods

### 2.1. Materials

Magnesium chloride (98%, Sigma Aldrich, UK), sodium hydroxide (99.6%, Fisher, UK), and fluorescein diacetate (FDA, 98%, Fluka, UK) were used as supplied. Poly(sodium 4-styrene sulfonate) sodium salt (PSS), average M.W. 70 kDa, and poly(allylamine hydrochloride) (PAH), average M.W. 15 kDa, were purchased from Sigma Aldrich, UK. Deionized water that was produced by a Milli-Q reverse osmosis system (Millipore, UK) was used in all of the experiments. *S.cerevisiae* (from Sigma-Aldrich, UK) was cultured, as follows. 10 mg of lyophilised *S.cerevisiae* (baker’s yeast) was hydrated in 10 mL of pre-autoclaved deionized water at room temperature. Afterwards, 1 mL of the hydrated yeast suspension was added to 100 mL of the pre-autoclaved YPD culture media (yeast extract, peptone, and dextrose) and incubated for 48 h at 30 °C [33]. *Escherichia coli*, which was sourced from Thermofisher (Invitrogen MAX Efficiency™ DH10B™), was kindly provided for our antibacterial tests by Prof. Rotchell’s group at the University of Hull, UK. *E. coli* was grown up using Luria-Bertani medium (LB medium) [34], which can be prepared from 1 g tryptone, 0.5 g yeast extract, and 1 g sodium chloride in 100 mL deionized water. Subsequently, these components were autoclaved for one hour at 1.5 bar at 125 °C. A few microlitres of the stock suspension of *E. coli* were dispersed in the autoclaved culture media next to the Bunsen burner once the culture media was cooled down to room temperature. The cultured *E. coli* was incubated with shaking at 25 °C for 48 h to yield 5×10^5^–1×10^6^ colony forming units per mL (CFU/mL). Flickinger’s group from North Carolina University, USA kindly provided *Chlamydomonas reinhardtii* (cc-124 strain). This microalgae culture was grown in Tris-Acetate-Phosphate (TAP) culture medium and incubated at a temperature of 30 °C. The *C. reinhardtii* culture media consisted of TAP salts (NH_4_Cl; MgSO_4_ · 7H_2_O; and, CaCl_2_ · 2H_2_O), phosphate buffer solution (PBS), and Hutner’s trace elements solution (EDTA disodium salt, ZnSO_4_ · 7H_2_O, H_3_BO_3_, MnCl_2_ · 4H_2_O, CoCl_2_ · 6H_2_O, CuSO_4_ · 5H_2_O, FeSO_4_ · 7H_2_O, (NH_4_)_6_Mo_7_O_24_ · 4H_2_O), all purchased from Sigma-Aldrich, UK. The microalgae batch was grown in TAP media at pH 7 while being illuminated for 72 h by a white luminescent lamp with a light intensity of 60 W m^−2^ under constant stirring on a magnetic stirrer.

### 2.2. Characterisation 

The Mg(OH)_2_NPs size distribution and the zeta potential were characterised by a Zetasizer Nano ZL instrument (Malvern, UK). A digital sonicator (Branson LTD) was utilized for dispersing the Mg(OH)_2_NPs samples at 40% amplitude for 15 min at 2.0 s ON/2.0 s OFF pulse time. The thermogravimetric analysis (TGA) of Mg(OH)_2_NPs was done using a Mettler Toledo TGA/DSC instrument under N_2_ atmosphere. The crystallite size of Mg(OH)_2_NPs at various temperatures was studied by X-ray diffraction (Siemens D5000 X-Ray Diffractometer at 0.15418 nm wavelength). A JEM 2011 (JEOL, Japan) Transmission Electron Microscopy (TEM) machine was used to characterise the particle size and the morphology of Mg(OH)_2_NPs on the microbial cells surface. The JEOL JSM-6480 LV SEM instrument was utilized for characterising the morphology of Mg(OH)_2_NPs with bacterial.

### 2.3. Synthesis of Mg(OH)_2_NPs

The Mg(OH)_2_NPs were prepared from magnesium chloride (MgCl_2_) as a source of magnesium ions and sodium hydroxide (NaOH) aqueous solutions. Precipitation was induced by a dropwise addition of 0.4 M NaOH into the 0.2 M MgCl_2_ solution under continuous stirring at different reaction temperatures (i.e., 25 °C, 50 °C, 75 °C and 100 °C) for 1 h. The white product was centrifuged and washed with copious amounts of high purity water and ethanol for the effective removal of impurities. The final product was dried at 80 °C for 24 h [6]. Aqueous dispersions of the Mg(OH)_2_NPs were then prepared by dispersing 0.025 g of the Mg(OH)_2_ sample in 100 mL deionized water by using a digital sonicator (Branson Ltd.) at 40% of the maximum power for 15 min at 2 s ON/2 s OFF pulse time. Figure S1 shows a schematic diagram of the synthesis method of Mg (OH)_2_NPs.

### 2.4. Preparation of Polyelectrolyte-Coated Mg(OH)_2_NPs

Polyelectrolyte-coated Mg(OH)_2_NPs were prepared by using the Mg(OH)_2_NPs synthesised at a reaction temperature of 75 °C. 50 mL of 1000 µg mL^−1^ Mg(OH)_2_NPs dispersion in deionized water were added dropwise to an equal amount of 50 mg mL^−1^ PSS (M.W. ~70 kDa) solution in 1 mM NaCl. The samples were washed three times by centrifugation for 1 h at 10,000 rpm to remove the excess of PSS after shaking for 1 h on orbital shaker. Finally, the PSS-coated Mg(OH)_2_NPs were re-dispersed in 50 mL deionized water [35] and the particle size and zeta potential measured by a Zetasizer Nano ZL instrument. To prepare the PAH-coated nanoparticles, the PSS-coated Mg(OH)_2_NP suspension was mixed dropwise with 50 mL of 50 mg mL^−1^ PAH (M.W. 15 kDa) that was dissolved in 1 mM NaCl solution. The mixture was shaken for 20 min and centrifuged three times at 10,000 rpm for one hour to yield Mg(OH)_2_NPs/PSS/PAH. 

### 2.5. Antimicrobial Assay of Polyelectrolyte-Coated Mg(OH)_2_NPs on S.cerevisiae

The effect of Mg(OH)_2_NPs on *S.cerevisiae* cells was examined after removing the cells from the culture media. 30 mL dispersion of *S.cerevisiae* cells were washed by centrifugation with deionized water three times, and then re-dispersed in 30 mL deionized water. 5 mL of *S.cerevisiae* cell dispersion in deionized water were incubated with 5 mL Mg(OH)_2_NPs aqueous suspension upon increasing total particle concentrations (0, 250, 500, 1000, 2500, and 5000 μg mL^−1^) for 10 min, 6 h, 12 h, and 24 h. 1 mL of each incubated *S.cerevisiae* sample was centrifuged at 3500 rpm for 4 min and washed with deionized water to remove the excess of Mg(OH)_2_NPs. The *S.cerevisiae* cells were re-suspended in 1 mL of deionized water, then two drops of 1 mM FDA solution in acetone were added to each sample and then mixed together for 15 min. After that, the samples were washed three times with deionized water by centrifugation at 3500 rpm for 4 min. Finally, the viability of the cells was examined by fluorescence microscopy and an automatic cell counter (Nexcelom Cellometer Auto X4 Fluorescence). Similar experiments were utilized to test the effect of Mg(OH)_2_NPs that were coated with polyelectrolytes on the viability of *S.cerevisiae.*

### 2.6. Antibacterial Assay of Polyelectrolyte-Coated Mg(OH)_2_NPs on E. coli

10 mL of the *E. coli* culture grown in LB medium was washed, centrifuged three times with deionized water at 5000 rpm for three minutes, and redispersed in 100 mL deionized water. Subsequently, 5 mL of the washed *E. coli* suspension were incubated with a series of 5 mL aliquots of aqueous dispersions of Mg(OH)_2_NPs of different concentrations (0, 250, 500, 750, 1000, 2500, 5000, and 6000 µg mL^−1^). After each incubation, 1 mL of each *E. coli* suspension sample was washed and re-suspended in 1 mL deionized water. Afterwards, 100 μL of culture media free *E. coli* bacteria were incubated with 100 μL of the BacTiter-Glo Microbial cell viability reagent in a white opaque 96-well solid flat bottom microplate, shaken for 30 s, and then incubated for five minutes at 25 °C. The relative luminance was measured as a function of incubation time to find out the cell viability upon incubation with different concentration of Mg(OH)_2_NPs. The same experiments were also repeated with polyelectrolyte-coated Mg(OH)_2_NPs. This was done by incubating an aliquot of the *E. coli* suspension (diluted 10 times) with Mg(OH)_2_NPs that were coated with poly(sodium-4-styrenesolfonate and poly(allylamine hydrochloride) for up to 24 h.

### 2.7. Antialgal Activity of Polyelectrolyte-Coated Mg(OH)_2_NPs to C. reinhardtii 

50 mL of *C. reinhardtii* culture were washed from the culture media three times by using deionized water and centrifugation, and finally re-dispersed in 30 mL deionized water. 5 mL of the washed *C. reinhardtii* cells were incubated with a series of 5 mL aliquots of aqueous Mg(OH)_2_NPs dispersions at different concentrations at 25 °C. A control sample of the same cells was treated in a similar way without any exposure to Mg(OH)_2_NPs. After that, 1 mL of the *C. reinhardtii* suspension was taken from each treated sample and washed with deionized water to remove the excess of nanoparticles by centrifugation at 3500 rpm for 4 min. The *C. reinhardtii* cells were re-suspended in 1 mL deionized water, and two drops of 1 mM FDA solution in acetone were added to each sample and mixed together for 15 min. After that, the samples were washed three times with deionized water by centrifugation at 3500 rpm for 4 min. Finally, the automatic cell counter was utilized to assay the microalgae cell viability. The same method was used to test the effect of Mg(OH)_2_NPs that were coated with PSS and PAH on the viability of *C. reinhardtii*, which were incubated at different particle concentrations for various exposure times.

### 2.8. SEM and TEM Sample Preparation Protocol for C. reinhardtii, S.cerevisiae and E. coli after Exposure to Bare- and Polyelectrolyte-Coated Mg(OH)_2_NPs 

The *C. reinhardtii, S.cerevisiae* and *E. coli* were washed by centrifugation and then fixed with 2.5% glutaraldehyde at room temperature for two hours in 0.1M cacodylate buffer pH 7.2 after incubation with the bare- or polyelectrolyte-coated Mg(OH)_2_NPs of various concentrations. These samples were then post-fixed in 1% osmium tetroxide for 1 h, and then dehydrated in a range of ethanol-water mixtures with an increasing ethanol content from 50 vol% up to 100 vol%, followed by a critical point drying. After incubation with Mg(OH)_2_NPs, the microbial cells were prepared for TEM imaging while using the following procedure. The cells were washed with deionized water to remove the excess of Mg(OH)_2_NPs by centrifugation at 500 rpm and then fixed in 2 wt% glutaraldehyde for one hour at room temperature. This was followed by a treatment with 1 wt% osmium tetroxide for one hour. Subsequently, the samples were incubated for one hour with 2.5% uranyl acetate and washed with aqueous ethanol solutions of increasing concentration, as described above. After standard dehydration, the microbial samples were embedded in fresh epoxy/Araldite at 60 °C for two days, left for two days at room temperature, and sectioned with an ultra-microtome. SEM and TEM imaged microbial samples before and after the nanoparticle treatment. 

### 2.9. Zeta Potential Measurements of the C. reinhardtii, S.cerevisiae and E. coli cells after treatment with Mg(OH)_2_NPs 

The changes of surface charge of *C. reinhardtii, S.cerevisiae,* and *E. coli* after exposure to Mg(OH)_2_NPs at different concentrations (0, 250, 500, 750, 1000, 2500, 5000, and 6000 µg mL^−1^) were determined by a Zetasizer Nano ZL instrument (Malvern, UK). The cells were removed from the excess of Mg(OH)_2_NPs in the aqueous phase by centrifugation, and then dispersed in deionized water. For each sample, an appropriate amount of undiluted solution was placed into the cuvette, and an average zeta potential value was obtained from three individual measurements. The solution media was deionized water in all of the zeta potential measurements.

### 2.10. MIC of Non-modified and PSS/PAH-coated Mg(OH)_2_NPs on Microbial Cells

The following protocol was used to determine the Minimal Inhibitory Concentration (MIC) of Mg(OH)_2_NPs and PSS/PAH-coated Mg(OH)_2_NPs on cells. A negative control of 100 µL of LB medium was added to the first line of wells of a 96 well plate. 50 µL of LB medium were added to the treatment wells and the positive bacteria control wells. A stock solution of Mg(OH)_2_NPs and PSS/PAH-coated Mg(OH)_2_NPs was created in fresh LB medium to a total volume of 10 mL. 50 µL of this formulation ware added to the first line of treatment wells, and serial diluted 1:2 across the 96 well plate, which ensured that it was mixed by pipetting up and down within each well. An overnight culture of *E. coli* was diluted into a sterilised 0.85% saline until an absorbance reading between 0.08 and 0.12 at 625 nm was obtained on a spectrophotometer (0.5 Mcfarland Standard). The saline diluted bacteria were further diluted 1:150 into LB (10 mL LB + 66.67 µL of bacteria in saline solution) yielding a 10 mL stock containing 5 × 10^5^–1 × 10^6^ cells per mL. 50 µL from this bacteria stock were added to each treatment and positive bacteria control wells, seeding with 2.5 × 10^4^–5 × 10^4^ cells per well. Each well contained a final volume of 100 µL, with decreasing concentrations of treatment on equal amounts of bacteria. The plate was incubated for 24 h at 37 °C. After incubation, 20 µL of resazurin solution were added to each well. The MIC was determined from the lowest concentration treatment, which inhibited growth.

## 3. Results and Discussion

### 3.1. Preparation and Characterisation of Mg(OH)_2_NPs

The mean hydrodynamic diameter and zeta potential of the Mg(OH)_2_NPs in deionized water were measured by the dynamic light scattering instrument (DLS) of suspensions that were prepared by dispersing 0.025 g of Mg(OH)_2_NPs sample in 100 mL of deionized water by a digital sonicator. The average hydrodynamic diameter of Mg(OH)_2_NPs was found to be 69 ± 10 nm and their zeta potential was +30 ± 6 mV, as shown in Figure S2 and Figure S3. The thermo gravimetric analysis (TGA) that was carried out between 50 and 1000 °C, as shown in Figure S4A, indicated that the Mg(OH)_2_ sample was stable up to 270 °C. Subsequently, the endothermic peak corresponding to the removal of the adsorbed water molecules took place between 270–300 °C, as also reported by other authors [36]. The major weight loss step has been found in the temperature range of 300–450 °C, which is due to the transition phase, corresponding to the decomposition of Mg(OH)_2_NPs to MgO. The TGA curve exhibits a total mass loss equal to 29.46%, which is slightly lower than the calculated mass loss (30.8%) that is attributed to the complete dehydroxylation process of Mg(OH)_2_. This result also agrees with previous studies [36,37,38]. An Energy dispersive X-ray Diffraction (EDX) analysis was carried out on the synthesised Mg(OH)_2_NPs to verify the elemental composition. The EDX data in Figure S4B confirm the presence of magnesium and oxygen signals in the Mg(OH)_2_NPs sample. The elemental analysis of the Mg(OH)_2_NPs yielded 36.59% of magnesium and 59.94% of oxygen, which indicates that the formed Mg(OH)_2_NPs were in its highly purified form and in agreement with previous studies [24].

We studied the effect of annealing temperature on the particle size of Mg(OH)_2_NPs for the synthesis that was done at various reaction temperature (25 °C, 50 °C, 75 °C, and 100 °C). Figure S4C shows the impact of the temperature of the reaction mixture on the size of Mg(OH)_2_NPs for one hour. Mg(OH)_2_NPs of lower average size were produced at 75 °C and 100 °C, while larger particles were created at 25 °C and 50 °C. Therefore, 75 °C and 100 °C were the optimal temperatures for the production of Mg(OH)_2_NPs. 

The zeta potential and particle size of the produced Mg(OH)_2_NPs was measured at pH in the range 3–12 and the results are shown in Figure S4D. The isoelectric point of the non-coated Mg(OH)_2_NPs was approximately at pH 11.7, i.e., the bare Mg(OH)_2_NPs are cationic at neutral pH. As it can be seen from Figure S4D, the zeta potential decreases and the particle size increases upon the increase of pH. The aggregation of Mg(OH)_2_NPs occurs above pH 8.5. The Fourier transform infrared spectroscopy (FTIR) spectrum of the Mg(OH)_2_NPs synthesised using a magnesium chloride solution at different reaction temperatures is shown in Figure S5 (ESI). The sharp and intense 3700 cm^−1^ FTIR peak corresponds to the Mg(OH)_2_ asymmetric O–H stretching. The band at 1400 cm^−1^ is due to the water O–H stretch. The strong and wide 570 cm^−1^ peak is due to Mg−O stretching. No other absorption peaks from impurities were detected. This result indicates that the Mg(OH)_2_ that was obtained had higher purity and it is also in agreement with previous studies [39,40]. 

### 3.2. Effect of the Precipitation Temperature on the Crystallite Size of the Synthesised Mg(OH)_2_NPs

**Figure 2:**
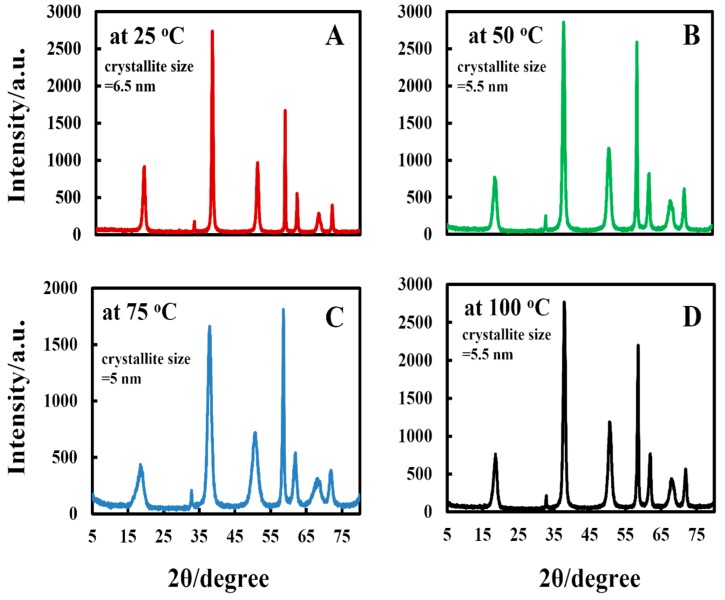
Comparison of the X-ray pattern of Mg(OH)_2_NPs precipitated at 25 °C (**A**), 50 °C (**B**), 75 °C (**C**), and 100 °C (**D**).

Figure 2 shows the XRD pattern of Mg(OH)_2_NPs samples that were obtained at various reaction temperatures 25 °C, 50 °C, 75 °C, and 100 °C while using magnesium chloride as a precursor. The diffraction peaks are in agreement with the hexagonal structure of Mg(OH)_2_NPs according to Joint Committee on Powder Diffraction Standards (JCPDS) Card No. 00-044-1482, which indicates that no apparent impurities are detected. The average crystallite size of Mg(OH)_2_NPs was calculated by using the Scherrer equation, *D* = *K*λ/βcosθ, were *D* is the crystallite size in nm, *K* is a dimensionless shape constant taken as 0.94, *2θ* is the diffraction angle, λ is the wavelength of the X-ray radiation (CuKα = 0.15406 nm), and β is the full width at the half-maximum (FWHM) of the diffraction peak.

### 3.3. Polyelectrolyte-Functionalized Mg(OH)_2_NPs

**Figure 3:**
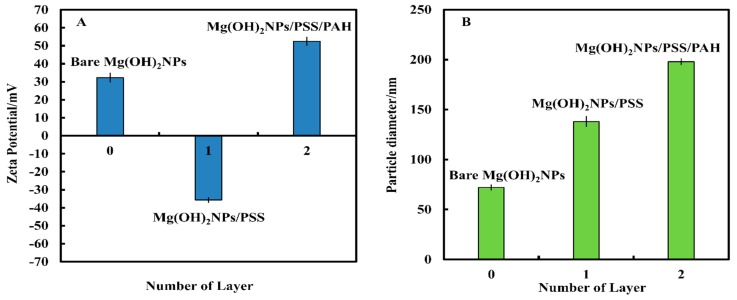
(**A**) The zeta potential and (**B**) particle size of bare-and polyelectrolyte-coated Mg(OH)_2_NPs.

We coated 69 nm Mg(OH)_2_NPs with two subsequent layers of PSS and PAH via the procedures explained above [35]. Figure 3A shows the zeta potential of the coated Mg(OH)_2_NPs as a function of the number of polyelectrolyte layers. The zeta potential of the Mg(OH)_2_NPs changed from approximately +30 mV to −36 mV for Mg(OH)_2_NPs/PSS. Further coating with PAH yielded positively charged Mg(OH)_2_NPs/PSS/PAH with a zeta potential of +51 mV. As expected, the particle surface charge alternates with the addition of the oppositely charged polyelectrolyte layer. Figure 3B shows that the coated NPs size increases after every subsequent polyelectrolyte coating due to partial aggregation.

### 3.4. Antimicrobial Assay of Bare Mg(OH)_2_NPs on S.cerevisiae, C. reinhardtii and E. coli

**Figure 4:**
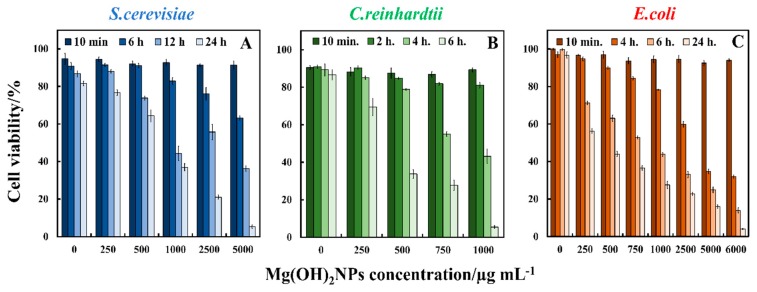
The antimicrobial activity of bare Mg(OH)_2_NPs on (**A**) *S.cerevisiae* (**B**) *C. reinhardtii* and (**C**) *E. coli* at various particle concentrations. The cells were incubated with the Mg(OH)_2_NPs at different periods of time shown.

We compared the antimicrobial activity of bare Mg(OH)_2_NPs on *C. reinhardtii*, *S. cerevisiae,* and *E. coli*, and Figure 4 shows the cell viability. We incubated samples of *S.cerevisiae* cells with dispersed Mg(OH)_2_NPs at different particle concentrations (0, 250, 500, 1000, 2500, and 5000 μg mL^−1^) for different periods of time, up to one day. Subsequently, an aliquot of every sample was taken to examine the number of viable *S.cerevisiae* cells while using an automatic cell counter by FDA cell viability assay. Figure 4A shows the percentage of viable *S.cerevisiae* cells at various incubation times (10 min., 6 h, 12 h, and 24 h). One can see that the percentage of viable *S.cerevisiae* after 10 min incubation is at the same level as in the untreated sample. After 6 h of incubation, no measurable antimicrobial effect is noticed up to 500 µg mL^−1^ Mg(OH)_2_NPs; however, antimicrobial activity is observed at 1000, 2500, and 5000 µg mL^−1^ Mg(OH)_2_NPs. After 12 h, the viability of *S.cerevisiae* sharply decreases at particle concentrations in the range 500–5000 µg mL^−1^. After one day of incubation, concentrations higher than 250 µg mL^−1^ bare Mg(OH)_2_NPs showed measurable antimicrobial activity towards *S.cerevisiae*. The optical microscopy examination of these samples suggests that the *S.cerevisiae* cells aggregate at a high particle concentration. Our results indicate a strong decline of the *S.cerevisiae* cell viability at concentrations above 1000 µg mL^−1^ bare Mg(OH)_2_NPs. Figure S15 (ESI) shows the same data, as in Figure 4A–C in CFU mL^−1^.

**Figure 5:**
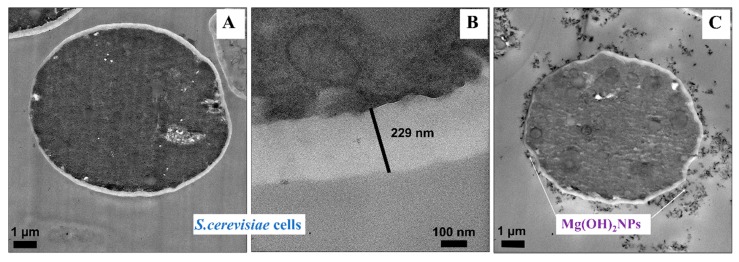
Transmission electron microscopy (TEM) images of *S. cerevisiae* after being incubated for one day with bare Mg(OH)_2_NPs: (**A**) A control sample without Mg(OH)_2_NPs. (**B**) A high-resolution TEM image of the *S. cerevisiae* wall without Mg(OH)_2_NPs. (**C**) *S. cerevisiae* cells incubated with 1000 µg mL^−1^ Mg(OH)_2_NPs showing the attachment of Mg(OH)_2_NPs to the outer cell surface.

The data in Figure 4A suggest that, at high concentrations of bare Mg(OH)_2_NPs, they electrostatically adhere to the negatively charged cell membranes, which subsequently kills the cells. The attachment of Mg(OH)_2_NPs to the cells was also examined by TEM imaging. Figure 5A,C show TEM images of the *S.cerevisiae* cells before and after treatment with 1000 µg mL^−1^ Mg(OH)_2_NPs solution for 24 h. Those are compared with the untreated samples of *S.cerevisiae* that are shown in Figure 5A,B. The TEM images show that, before the treatment (Figure 5A), the membrane of the *S.cerevisiae* cells is regular and smooth, the treatment with 1000 µg mL^−1^ Mg(OH)_2_NPs leads to a significant accumulation of Mg(OH)_2_NPs on the external wall of *S.cerevisiae* at such a high particle concentration (Figure 5C).

We also confirmed these results by EDX of the treated *S. cerevisiae* cells, which showed the presence of Mg on the outer part of the cell membrane (Figure S6, ESI). Although the exposure of the *S. cerevisiae* cells to bare Mg(OH)_2_NPs at concentration 1000 µg mL^−1^ caused a cytotoxicity effect (Figure 5C), it did not cause an internalisation of Mg(OH)_2_NPs, as the cell wall of *S. cerevisiae* cells is very thick (200 nm, Figure 5B) when compared to other microbial cells. We envisage two probable mechanisms for the antimicrobial effect of Mg(OH)_2_NPs on yeast. There is a significant accumulation of Mg(OH)_2_NPs on the cell membranes due to their cationic nature at neutral pH. As these particles have very irregular morphology and they consist of smaller crystallites, their adhesion to the cell membrane in large amounts can potentially cause its dislocation and cracking. Local damage of the membrane may lead to cell viability loss although we do not observe a straight permeation of Mg(OH)_2_NPs to the *S.cerevisiae* wall. Another possible mechanism of membrane damage can be caused by the counter-ion atmosphere of the Mg(OH)_2_NPs, which consists of highly concentrated hydroxyl ions (OH^-^) of very high local pH which can cause lipid hydrolysis on the membrane surface and killing the cell. Some of these mechanisms have been commented on by other authors for uncoated Mg(OH)_2_NPs [17,24,28].

**Figure 6:**
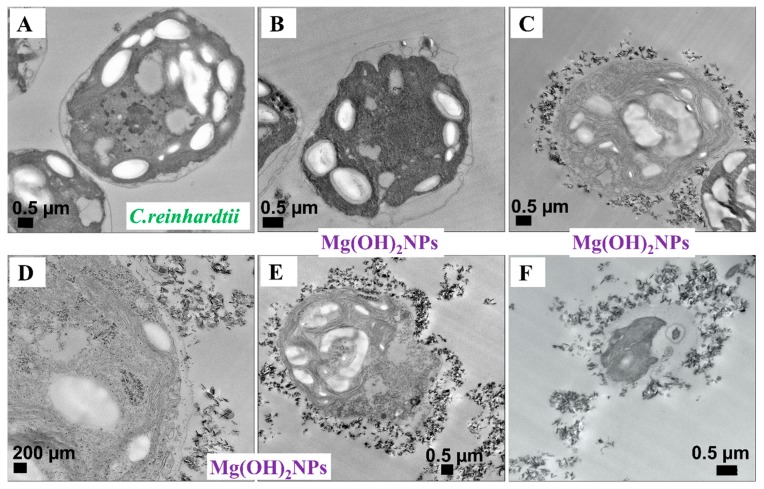
TEM images of *C. reinhardtii* after being exposed to 0, 250, 750, 1000 and 5000 µg mL^−1^ Mg(OH)_2_NPs for six hours (fixed, embedded in resin and sectioned). (**A**) An untreated sample without Mg(OH)_2_NPs; (**B**) *C. reinhardtii* treated with 250 μg mL^−1^ Mg(OH)_2_NPs; (**C**) and (**D**) *C. reinhardtii* incubated with 750 μg mL^−1^ Mg(OH)_2_NPs at different magnifications. (**E**) and (**F**) *C. reinhardtii* treated with 1000 μg mL^−1^ and 5000 μg mL^−1^ Mg(OH)_2_NPs, respectively.

We also examined the antimicrobial activity of Mg(OH)_2_NPs towards *C. reinhardtii* under similar conditions for various exposure times, as shown in Figure 4B. At a 10 min exposure time, all of the *C. reinhardtii* were viable at the similar level as the untreated sample. After two hours of incubation, the *C. reinhardtii* viability declined for Mg(OH)_2_NPs concentrations from 250 µg mL^−1^ to 1000 µg mL^−1^. After 4 h, the *C. reinhardtii* viability was reduced to 40% at 1000 µg mL^−1^ of Mg(OH)_2_NPs, while, after 6 h, it sharply declined for 250 µg mL^−1^ to 1000 µg mL^−1^ concentrations of Mg(OH)_2_NPs, which led to the complete loss of cell viability at concentrations above 750 µg mL^−1^ Mg(OH)_2_NPs. Figure 6 shows TEM images of *C. reinhardtii* exposed to Mg(OH)_2_NPs at various concentrations. TEM images of the *C. reinhardtii* after 6 h of incubation with Mg(OH)_2_NPs indicate the localization of the Mg(OH)_2_NPs with respect to the cell membrane. One can see that the outer cell wall of *C. reinhardtii* obtained a thick layer of associated NPs after treatment with 750 µg mL^−1^, 1000 µg mL^−1^ and 5000 µg mL^−1^ concentrations of Mg(OH)_2_NPs (Figure 6C–F). The internalization of Mg(OH)_2_NPs in the *C. reinhardtii* was not observed, even at 5000 μg mL^−1^ Mg(OH)_2_NPs, as shown in Figures S7 and S8. The EDX shows the absence of Mg in the inside of *C. reinhardtii,* but confirms its presence on the outer wall. 

Figure 4C shows the results for the effect of bare Mg(OH)_2_NPs towards *E. coli* at various incubation times. The data demonstrate that the bare Mg(OH)_2_NPs have excellent antimicrobial effects on the *E. coli* at 6000 µg mL^−1^ for one day. The *E. coli* viability sharply decreases for treatment with 6000 µg mL^−1^ Mg(OH)_2_NPs after four hours of exposure. The viability decreases further after six hours and, after one day, it resulted in approximately 97% loss of viability. Past research suggested that the antibacterial effect might be credited to multiple factors: (i) the cellular internalization of NPs where they could potentially interfere with the bacterial DNA and cellular organelles [41,42,43,44,45]; (ii) immediate contacts with the bacterial cell wall [23,24]; and, (iii) the increased local dissolution of metal ions of the nanoscale metal oxide [46,47]. Usually, the antimicrobial impact is dependent on the size of the nanoparticles and a better antimicrobial effect is achieved with smaller nanoparticles [17,48,49,50].

**Figure 7:**
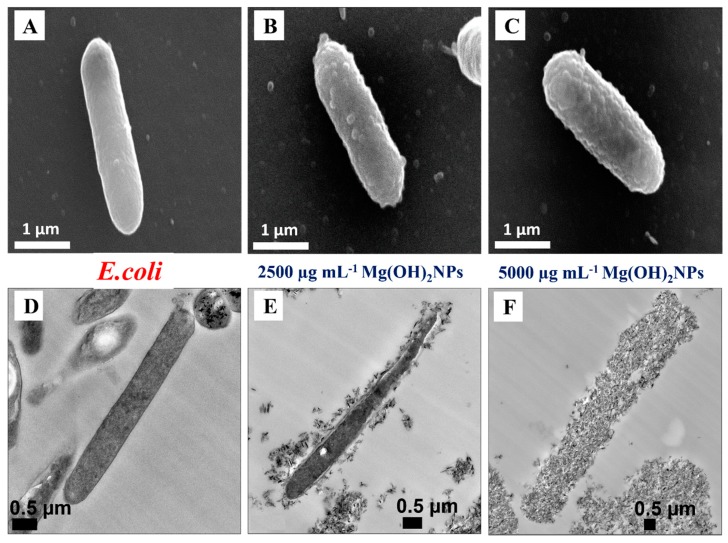
Scanning electron microscopy (SEM) and TEM images of *E. coli* after being incubated for 24 h with a suspension of bare Mg(OH)_2_NPs: (**A**) SEM and (**D**) TEM images of an untreated sample. (**B**) SEM and (E) TEM images of *E. coli* incubated with 2500 µg mL^−1^ Mg(OH)_2_NPs. (**C**) SEM and (**F**) TEM images of *E. coli* incubated with 5000 µg mL^−1^ Mg(OH)_2_NPs.

The Mg(OH)_2_NPs with the smallest size (about 70 nm) had the highest antimicrobial activity. We undertook TEM imaging and EDX analysis to examine the location of magnesium in the *E. coli* after treating them with non-coated Mg(OH)_2_NPs. Magnesium was not detected by the EDX in many randomly selected regions inside the *E. coli,* but it was primarily found on the outer side of the cell wall, as shown in Figure S9 (ESI). This showed that the dissolved magnesium ions and Mg(OH)_2_NPs did not go to the inside of *E. coli*. Nevertheless, evident changes of the *E. coli* cell structure were seen after incubation with Mg(OH)_2_NPs. Figure 7D shows the images of untreated *E. coli,* where the bacteria have preserved the integrity of their cell walls. After incubation with 2500 µg mL^−1^ (7E) and 5000 µg mL^−1^ (7F) Mg(OH)_2_NPs for one day, the cell profile became fussy and the walls of *E. coli* appear as disintegrated. Therefore, the antimicrobial activity of Mg(OH)_2_NPs might be expressed more via their adsorption on the outer side of the cell wall, rather than through internalization in the cell, which leads to the decay of the cell walls of *E. coli*. Furthermore, SEM imaging was used to study the presence of Mg(OH)_2_NPs on the surfaces of the bacteria. Figure 7A–C show *E. coli* samples after being treated with 2500 µg mL^−1^ and 5000 µg mL^−1^ Mg(OH)_2_NPs for one day. They indicate that the cell wall has a build-up of a dense layer of nanoparticles. Moreover, EDX indicated that the samples contain magnesium, and confirmed that the Mg(OH)_2_NPs have the ability to adhere on the bacterial cell wall with occasional penetration on the inside. Consequently, the *E. coli* lack of viability is associated with the compromised integrity of bacteria walls, which is seen by SEM and TEM for these samples. This is consistent with the mechanisms outlined above which indicates that the antimicrobial action of Mg(OH)_2_NPs on the cells is likely to be due to the cationic character of Mg(OH)_2_NPs that adsorb on the negatively charged bacterial cell wall by electrostatic attraction. The adsorbed Mg(OH)_2_NPs disrupt the integrity of the bacterial cell wall, which then increases its permeability and kills the bacteria.

### 3.5. Zeta Potential Measurements of Cells after Treatment with Mg(OH)_2_NPs

**Figure 8:**
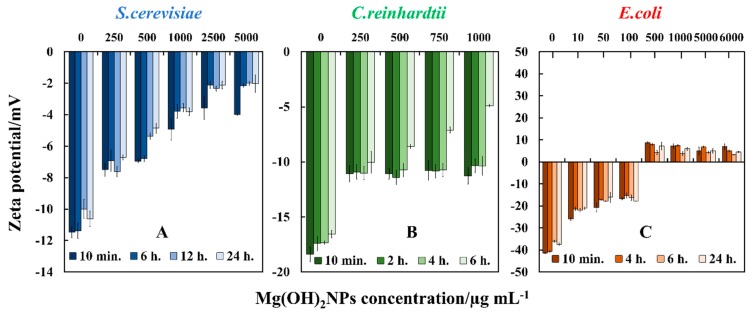
The zeta potential of cells treated with different concentrations of Mg(OH)_2_NP suspensions at various incubation times: (**A**) *S.cerevisiae,* (**B**) *C. reinhardtii,* and (**C**) *E. coli* cells.

We further explored the effect of the particles attachment on the outer cell wall, as it may play a significant role on their antimicrobial action [17,24,28]. We studied the zeta potential of the *S.cerevisiae*, *C. reinhardtii,* and *E. coli* after treatment with Mg(OH)_2_NPs in solution. The cells were incubated with Mg(OH)_2_NPs suspensions at different particle concentrations. Subsequently, an aliquot of every suspension was taken to examine the cells average zeta potential value by a Zetasizer instrument. We found that Mg(OH)_2_NPs have an average zeta potential of +30 ± 6 mV. Upon incubation with bare Mg(OH)_2_NPs, the *S.cerevisiae* cells, which are negatively charged (zeta potential of −12 ± 5 mV), still were shown to be negative, but significantly reduced by magnitude zeta potential due to a build–up of cationic NPs, as shown in Figure 8A. Note that the zeta potential of threated cells does not vary much with the duration of the treatment.

The *C. reinhardtii* cells that have a negative zeta potential of −18 ± 5mV also reduced their zeta potential by magnitude after treatment with the cationic Mg(OH)_2_NPs, but did not charge reverse, even at high particle concentrations, as presented in Figure 8B. Figure 8C shows the impact of bare Mg(OH)_2_NPs on the *E. coli* zeta potential. *E. coli* cells, which carried negative charge (zeta potential −41±5 mV), remained negatively charged when treated with up to 100 µg mL^−1^ Mg(OH)_2_NPs. At higher Mg(OH)_2_NPs concentration, the zeta potential of *E. coli* cells turned positive when exposed to 500 µg mL^−1^ to 6000 µg mL^−1^ Mg(OH)_2_NPs. These results show that the adhesion of Mg(OH)_2_NPs to cells might indeed be primarily driven by electrostatic interactions [17,51]. It can be concluded that the positive charge of Mg(OH)_2_NPs has high impact on the adsorption of particles on the cells membrane. The SEM and TEM images confirmed this (Figure 6).

### 3.6. Antimicrobial Assay of Polyelectrolyte-Coated Mg(OH)_2_NPs on S.cerevisiae, C. reinhardtii, and E. coli

**Figure 9:**
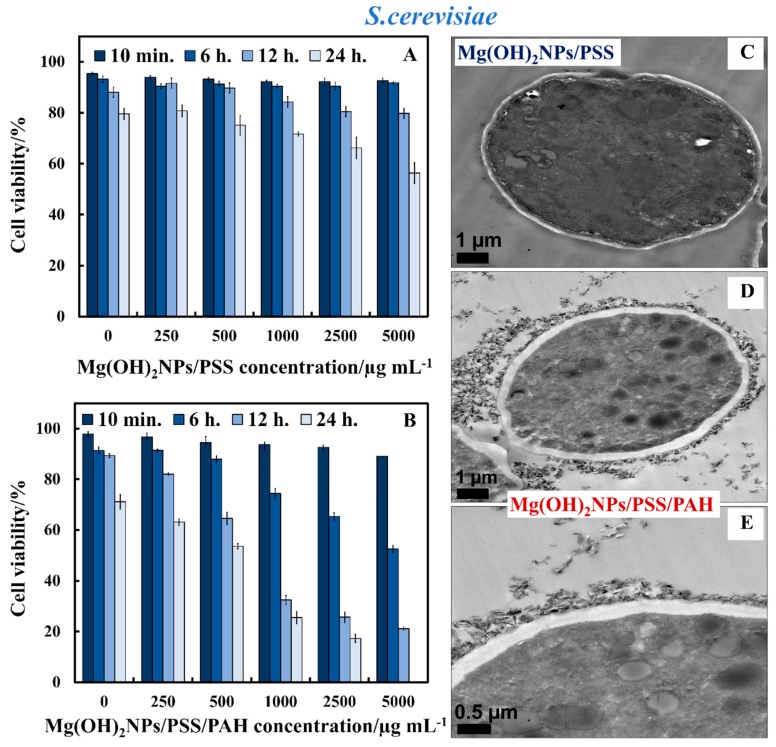
*S. cerevisiae* cell viability as a function of nanoparticle concentration after incubation for up to 24 h with (**A**) Mg(OH)_2_NPs/PSS and (**B**) Mg(OH)_2_NPs/PSS/PAH. TEM images of *S. cerevisiae* cells incubated for 24 h with (**C**) Mg(OH)_2_NPs/PSS and (**D**,**E**) Mg(OH)_2_NPs/PSS/PAH at different magnifications.

We studied the antimicrobial activity of Mg(OH)_2_NPs that were coated with multilayers of polyelectrolytes on *S.cerevisiae, C. reinhardtii,* and *E. coli*. We functionalized Mg(OH)_2_NPs with PSS and PAH and compared their antimicrobial effect with that of the bare Mg(OH)_2_NPs. The aqueous suspensions of *S.cerevisiae* were incubated with Mg(OH)_2_NPs/PSS and Mg(OH)_2_NPs/PSS/PAH suspensions at various particle concentrations (0, 250, 500, 1000, 2500, and 5000 μg mL^−1^) for up to one day. The results represented in Figure 9A show that the anionic Mg(OH)_2_NPs/PSS have a lower antimicrobial activity on *S.cerevisiae* when compared to the cationic bare Mg(OH)_2_NPs (c.f. Figure 4A). No change in the *S.cerevisiae* viability was registered for Mg(OH)_2_NPs/PSS, even at high particle concentrations, at incubating times up to six hours. The same treatment with the cationic Mg(OH)_2_NPs/PSS/PAH showed a significant antimicrobial activity on *S.cerevisiae* at particle concentrations of 1000, 2500, and 5000 μg mL^−1^, as shown in Figure 9B. A very strong effect of the Mg(OH)_2_NPs/PSS/PAH on *S.cerevisiae* viability was observed upon their incubation with high particle concentrations of 5000 μg mL^−1^ for up to 24 h. In contrast, upon incubation with the anionic Mg(OH)_2_NPs/PSS at high particle concentrations of 5000 μg mL^−1^, we observed a moderate impact on *S.cerevisiae* viability for up to one day of incubation (Figure 9A). Figure S12 compares the anti-yeast activity bare Mg(OH)_2_NPs with Mg(OH)_2_NPs/PSS and Mg(OH)_2_NPs/PSS/PAH as a function of the nanoparticle concentration for 24 h of exposure. 

Hence, by coating the Mg(OH)_2_NPs with an outer layer of anionic polyelectrolyte, their antimicrobial activity is significantly decreased because of the electrostatic repulsion between the anionic Mg(OH)_2_NPs/PSS and the anionic surface of *S. cerevisiae* cells. Figure 9C–E show TEM images of *S. cerevisiae* cells after their incubation with Mg(OH)_2_NPs that were coated with PSS and PAH layers.

**Figure 10:**
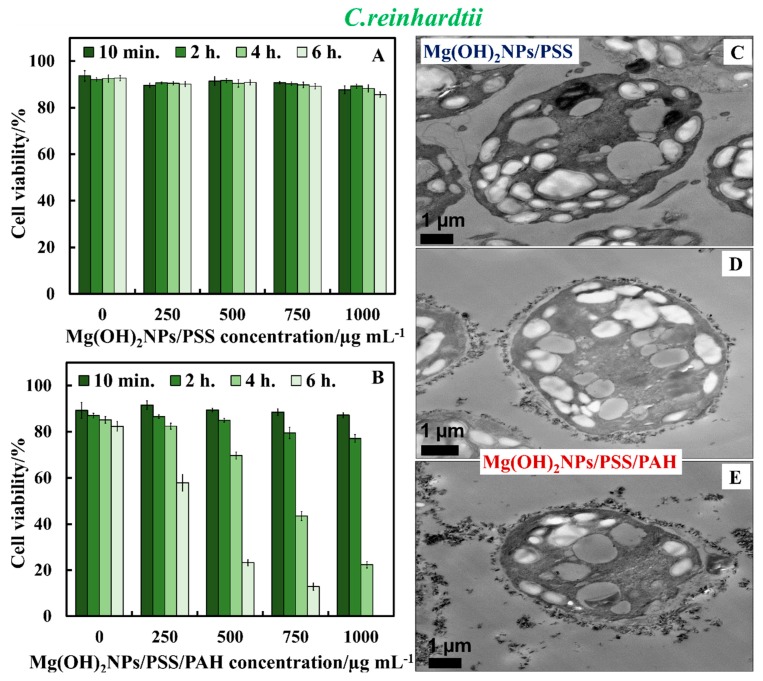
*C. reinhardtii* cell viability as a function of nanoparticle concentration after incubation for up to 6 h with (**A**) Mg(OH)_2_NPs/PSS and (**B**) Mg(OH)_2_NPs/PSS/PAH. TEM images of *C. reinhardtii* after being incubated for 6 h with (**C**) 1000 μg mL^−1^ Mg(OH)_2_NPs/PSS, (**D**) 750 μg mL^−1^ Mg(OH)_2_NPs /PSS/PAH, and (**E**) 1000 μg mL^−1^ of Mg(OH)_2_NPs /PSS/PAH.

The TEM image in Figure 9C indirectly confirms the lack of nanoparticle accumulation due to the electrostatic repulsion among the anionic Mg(OH)_2_NPs/PSS and the negatively charged *S. cerevisiae* cell wall. Figure 9D,E show the great accumulation of Mg(OH)_2_NPs/PSS/PAH on the cell walls, which corresponds to a much higher activity towards *S. cerevisiae*. One can conclude that coating Mg(OH)_2_NPs with PSS as an external layer significantly diminishes their ability to attach on the treated cells, as shown in Figure 9C. *S. cerevisiae* cell viability tests revealed that Mg(OH)_2_NPs/PSS were much less effective in killing the cells than the Mg(OH)_2_NPs/PSS/PAH or bare Mg(OH)_2_NPs, which strongly accumulate on the cell membrane due to electrostatic attraction. These results were also supported by the TEM images of *S. cerevisiae*. Figure S16 shows the same data as Figure 9A,B in CFU ml^−1^. Figure 10A,B compares the antimicrobial activity of multilayer-coated Mg(OH)_2_NPs with PSS and PAH polyelectrolytes at various NPs concentrations on the *C. reinhardtii*. Figure 10A shows that, for incubating times of up to 6 h, no measurable variation in the *C. reinhardtii* cell viability was detected for Mg(OH)_2_NPs/PSS, even at high particle concentrations. However, at similar conditions, the cationic Mg(OH)_2_NPs/PSS/PAH displayed a marked antimicrobial activity on *C. reinhardtii*, even at 250 µg mL^−1^. A very strong effect of the Mg(OH)_2_NPs/PSS/PAH on the *C. reinhardtii* cells viability was observed for exposure times of up to six hours at 1000 µg mL^−1^ particle concentrations (Figure 10B). One can conclude that, by coating the Mg(OH)_2_NPs with an external layer of anionic polyelectrolyte, their antimicrobial activity decreased for both *S. cerevisiae* and *C. reinhardtii,* because of the electrostatic repulsion between the Mg(OH)_2_NPs/PSS and the cells walls. Figure 10C–E confirm this hypothesis with TEM images of *C. reinhardtii* exposed into the polyelectrolyte-coated Mg(OH)_2_NPs. We also conducted similar tests with Gram-negative bacteria (*E. coli*) and polyelectrolyte-coated Mg(OH)_2_NPs when the bacterial cells were removed from their culture media. Figure S17 (ESI) shows the same data as Figure 10A,B in CFU mL^−1^. Figure S13 compares the anti-yeast activity bare Mg(OH)_2_NPs with Mg(OH)_2_NPs/PSS and Mg(OH)_2_NPs/PSS/PAH as a function of the nanoparticle concentration for 24 h of exposure. 

**Figure 11:**
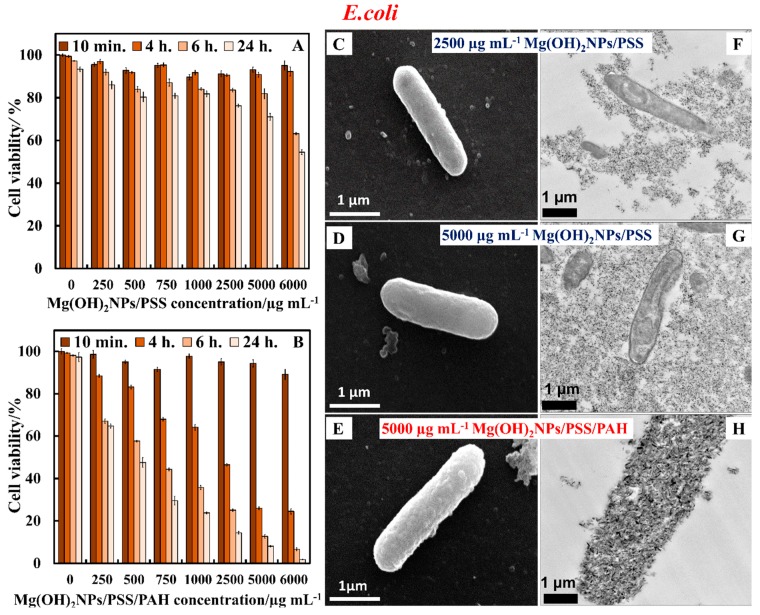
The *E. coli* cell viability after treatment with (**A**) Mg(OH)_2_NPs/PSS and (**B**) Mg(OH)_2_NPs/PSS/PAH for various incubation times as a function of the NPs concentration. (**C**) SEM and (**F**) TEM images of *E. coli* after incubation with 2500 µg mL^−1^ Mg(OH)_2_NPs/PSS; (**D**) SEM and (**G**) TEM images of *E. coli* after incubation with 5000 µg mL^−1^ Mg(OH)_2_NPs/PSS; (**E**) SEM and (**H**) TEM images of *E. coli* after incubation with 5000 µg mL^−1^ Mg(OH)_2_NPs/PSS/PAH. The cells were removed from the particle suspension before the sample preparation for TEM and SEM imaging.

Figure 11A,B show the effect of polyelectrolyte multilayer-coated Mg(OH)_2_NPs against *E. coli*. Similarly to *S. cerevisiae* and *C. reinhardtii,* we found no pronounced antibacterial effect of Mg(OH)_2_NPs/PSS on *E. coli* for various exposure times. Figure 11A shows that the antibacterial activity of Mg(OH)_2_NPs/PSS against *E. coli* is also much lower than the one of the bare Mg(OH)_2_NPs. We envisage that this result is due to a similar decrease of the NPs accumulation on the bacterial cell wall after the functionalization of the Mg(OH)_2_NPs with an anionic PSS layer (see Figure 11C,D,F,G). The subsequent deposition of a cationic polyelectrolyte layer of PAH, yields Mg(OH)_2_NPs/PSS/PAH, which showed excellent antibacterial properties against *E. coli*, as seen in Figure 11B. Note that the PAH-coated NPs have even stronger antibacterial activity than the uncoated Mg(OH)_2_NPs towards *E. coli*. Hence, the antibacterial activity of the polyelectrolyte coated Mg(OH)_2_NPs appears to alternate with their surface charge. Figure S18 (ESI) shows the same data as in Figure 10A,B in CFU mL^−1^.

The *E. coli* Gram-negative cell wall is composed of an organized triple membrane that contains a thin inner layer of peptidoglycan between an outer membrane consisting of porins [41], phospholipids molecules, lipopolysaccharides (LPS), lipoproteins, surface proteins, and a cytoplasmic membrane consisting of phospholipids molecules and porins (see Figure S10) [41]. Figure 11C–H show the SEM and TEM images of *E. coli* after treatment for 24 h with Mg(OH)_2_NPs coated with a single layer of PSS and ones with additional layer of PAH. Note that there are a very few Mg(OH)_2_NPs/PSS that are attached to the bacteria, as shown in Figure 11C,D,F,G. On the other hand, we found a significant accumulation of Mg(OH)_2_NPs/PSS/PAH onto the surface of the bacteria, as shown in Figure 11E,H. These SEM and TEM images are consistent with the antibacterial activity profile of the polyelectrolyte-coated Mg(OH)_2_NPs against *E. coli,* as reported in Figure 11B. It can be argued that the weak attachment of the anionic particles Mg(OH)_2_NPs/PSS to the bacteria, as supported via the SEM and TEM images, causes little damage of the bacteria wall. Figure 7B,C,E,F for the bare Mg(OH)_2_NPs and Figure 11E,H for the Mg(OH)_2_NPs/PSS/PAH, show that there is a substantial build-up of cationic NPs (uncoated and PAH-coated Mg(OH)_2_NPs) onto the anionic bacterial cell surface, which corresponds to a greater local increase of the NPs concentration that successively disrupts the bacteria. Figure S14 compares the anti-yeast activity bare Mg(OH)_2_NPs with Mg(OH)_2_NPs/PSS and Mg(OH)_2_NPs/PSS/PAH as a function of the nanoparticle concentration for 24 h of exposure. We also examined the antibacterial activity of MgCl_2_ solutions of various concentrations for 24 h on *E. coli* in order to investigate if this is due to higher local concentration of Mg^2+^ ions, where the bacterial cells were extracted from the culture media in a similar processes, as explained above for the Mg(OH)_2_NPs treatment with *E. coli*. We found that MgCl_2_ did not have significant antibacterial action when compared to the Mg(OH)_2_NPs, even at high concentrations, as shown in Figure S11 (ESI). The effect of the pH on the bacterial cell viability is similar—we found that the incubation of bacteria with NaOH solution of pH 10.4 (corresponding to the pH of bare Mg(OH)_2_NPs suspension) did not produce a comparable effect. Hence, the analysis of these results suggests that magnesium ions in the Mg(OH)_2_NPs and the basic pH of 10.4 is unlikely to be responsible for the killing of *E. coli* [28]. The most likely explanation is the rough surface morphology of the clustered Mg(OH)_2_NPs, which, when electrostatically attracted towards the cell membrane, cause membrane disruptions that kill the bacteria.

Note that neither Mg^2+^ ions nor reactive oxygen species (ROS) generation can explain the antimicrobial properties of the Mg(OH)_2_NPs. The solubility of Mg(OH)_2_NPs is too low for the free Mg^2+^ ions to have any measurable cytotoxic effect, as their concentration is limited by the solubility product of Mg(OH)_2_NPs (1.8 × 10^−11^ M^3^). The Mg(OH)_2_NPs are not a photoactive material, which means that it is not producing ROS upon illumination. Hence, the antimicrobial effect is likely coming from the surface roughness of the Mg(OH)_2_NPs, which electrostatically stick onto the negatively charged microbial cells due to their cationic character and pierce their cell membrane. The effect is also amplified by the high concentration of OH- ions in their electric double layers, which they bring in close contact with the microbial cell surface upon adhesion. This can potentially lead to partial hydrolysis of the lipids in their cell membranes and cell death.

We also determined the MIC of free Mg(OH)_2_NPs and PAH-coated Mg(OH)_2_NPs on *E. coli*, *S. cerevisiae,* and *C. reinhardtii*. We found that, at the same conditions, the MIC of the PAH-coated Mg(OH)_2_NPs is two times lower than that of free Mg(OH)_2_NPs (see Table 1).

### 3.7. Effect of the bare Mg(OH)_2_NPs and PSS/PAH-coated Mg(OH)_2_NPs on human cells

Figure S19 (ESI) shows the cytotoxicity assay of the bare Mg(OH)_2_NPs and Mg(OH)_2_NPs/PSS/PAH on human embryonic kidney cells (HEK 293 cell line) for up to 24 h of exposure. The cells were removed from the original culture media and then transferred to PBS before exposure to the nanoparticles. This was done to avoid the adsorption of the media components on the particles. Both runs were done at the varying overall nanoparticles concentration and at different incubation times. One can see a very small effect on the presence of free Mg(OH)_2_NPs and Mg(OH)_2_NPs/PSS/PAH on the cells viability over a period of up to 24 h. Note that the control sample of HEK 293 cells has lost a minor fraction of their viability over this period of time due to the removal of the culture media. One can conclude that the nanoparticle does not measurably impact the HEK 292 cell viability up to 2500 µg mL^−1^. However, the effect on yeast, algae, and *E. coli* is very significant at these concentrations of free Mg(OH)_2_NPs and Mg(OH)_2_NPs/PSS/PAH—see Figure 4, Figure 9B, Figure 10B and Figure 11B, respectively. Therefore, one may conclude that the Mg(OH)_2_NPs show excellent biocompatibility with this human cell line. More research will be conducted in the future regarding the effects of the nanoparticles on different type of other cell lines.

## 4. Conclusions

When compared with other inorganic nanoparticles that were studied in the literature, Mg(OH)_2_NPs have high antimicrobial activity at moderate to high particle concentrations. However, the Mg(OH)_2_NPs have great application potential as a new antimicrobial agent, since Mg(OH)_2_ is a nontoxic material and has been broadly used in medical industries and food. Here, we studied various ways to control the antimicrobial activity and cytotoxicity of a range of bare and surface-modified Mg(OH)_2_NPs on three different types of microbial cells: microalgae, yeast, and bacteria. The antimicrobial activity of the Mg(OH)_2_NPs on *S. cerevisiae*, *C. reinhardtii,* and *E. coli* was examined. This work suggests that bare Mg(OH)_2_NPs are effective antimicrobial agents. The results from TEM and SEM analysis showed that the direct contact between the Mg(OH)_2_NPs and the cell membrane of *S. cerevisiae*, *C. reinhardtii,* and *E. coli* is very important for their effective antimicrobial action. A series of polyelectrolyte-coated Mg(OH)_2_NPs were likewise synthesised while using the layer by-layer technique and their antimicrobial activity towards *S. cerevisiae*, *C. reinhardtii,* and *E. coli* was compared with that of bare Mg(OH)_2_NPs in order to evaluate the role of the surface coating. It was discovered that the antimicrobial activity of the coated Mg(OH)_2_NPs alternates with their surface charge. The anionic nanoparticles (Mg(OH)_2_NPs/PSS) have much lower antibacterial activity than the cationic ones (Mg(OH)_2_NPs/PSS/PAH and bare Mg(OH)_2_NPs). We also show that Mg(OH)_2_NPs/PSS/PAH and bare Mg(OH)_2_NPs have only very minor impact on selected human cells line (HEK293), which implies good biocompatibility. This can bring important insights as to how the antmicrobial properties of Mg(OH)_2_NPs and other inorganic nanoparticles can be controlled by designing nanoparticle surface coatings that promote their adhesion to the microbial cell walls, as well as by taking into account the nanoparticles surface morphology.

## Figures and Tables

**Table 1:** Minimum inhibitory concentration (MIC) of bare Mg(OH)_2_NPs and PSS/PAH-coated Mg(OH)_2_NPs against *C. reinhardtii*, *S. cerevisiae* and *E. coli*.

	Mg(OH)_2_NPs	PSS/PAH-coated Mg(OH)_2_NPs
	MIC	MIC
*C. reinhardtii*	1000 µg mL^−1^	750 µg mL^−1^
*S.cerevisiae*	5000 µg mL^−1^	2500 µg mL^−1^
*E. coli*	5000 µg mL^−1^	2500 µg mL^−1^

## Supplementary Materials

The following are available online at https://www.mdpi.com/2313-7673/4/2/41/s1, Figure S1. A schematic overview summarizing the synthesis method of Mg(OH)_2_NPs, Figure S2. Particle size of Mg(OH)_2_NPs made from magnesium chloride at 75°C, Figure S3. The zeta potential of Mg(OH)_2_NPs made from magnesium chloride at 75°C, Figure S4. (A) Thermal gravimetric analysis pattern of Mg(OH)_2_NPs powder. (B) The EDX spectra of the uncoated Mg(OH)_2_NPs. (C) The impact of reaction temperature on the size of the produced Mg(OH)_2_NPs. (D) Variations in particle size and zeta potential of Mg(OH)_2_NPs suspensions with pH, Figure S5. FTIR spectra of the as prepared Mg(OH)_2_NPs at different reaction temperatures, Figure S6. EDX diagram of *S. cerevisiae* cells at 1000 µg mL^−1^, Figure S7. EDX chart of the *C. reinhardtii* with Mg(OH)_2_NPs at 1000 µg mL^−1^, Figure S8. EDX chart of the *C. reinhardtii* with Mg(OH)_2_NPs at 5000 µg mL^−1^, Figure S9. EDX diagram of *E. coli* cells incubated with Mg(OH)_2_NPs at 2500 µg mL^−1^ and 5000 µg mL^−1^, Figure S10. Schematic overview of the bacterial cell wall, Figure S11. The antibacterial impact of various concentration of MgCl_2_ towards *E. coli* for various exposure times, Figure S12. *S. cerevisiae* cell viability after incubation as a function of nanoparticle concentration for up to 24 h with uncoated and polyelectrolyte-coated Mg(OH)_2_NPs; Figure S13. The antialgal activity of uncoated and polyelectrolyte-coated Mg(OH)_2_NPs, Figure S14. Relationship between the antibacterial efficiency of uncoated and polyelectrolyte-coated Mg(OH)_2_NPs on the viability of *E. coli*., Figure S15. Colony forming unit (CFU) count of bare Mg(OH)_2_NPs on (A) *S. cerevisiae* (B) *C. reinhardtii* and (C) *E. coli* at various particle concentrations, Figure S16. Colony forming unit (CFU) count of *S. cerevisiae* as a function of nanoparticle concentration after incubation for up to 24 hours with (A) Mg(OH)_2_NPs/PSS and (B) Mg(OH)_2_NPs/PSS/PAH, Figure S17. Colony forming unit (CFU) count of *C. reinhardtii* as a function of nanoparticle concentration after incubation for up to 6 h with (A) Mg(OH)_2_NPs/PSS and (B) Mg(OH)_2_NPs/PSS/PAH, Figure S18. Colony forming unit (CFU) count of *E. coli* after treatment with (A) Mg(OH)_2_NPs/PSS and (B) Mg(OH)_2_NPs/PSS/PAH for various incubation times as a function of the NPs concentration, Figure S19. Comparison of the cell viability of human embryonic kidney cells (HEK 293 cell line) upon incubation as a function of nanoparticle concentration for up to 24 h with bare Mg(OH)_2_NPs and Mg(OH)_2_NPs/PSS/PAH, Figure S20. Antibacterial activity of SiO_2_NPs at various concentrations on *E. coli*., Figure S21. The anti-yeast, anti-algal and antibacterial activity of free PAH at various concentrations on (A) *S. cerevisiae*, (B) *C. reinhardtii* (C) *E. coli*.
