# Supplementary material for: Controlling the Antimicrobial Action of Surface Modified Magnesium Hydroxide Nanoparticles
Source: Biomimetics (Basel). 2019 Jun 25;4(2):41. doi: 10.3390/biomimetics4020041 (PMC6631741; doi:10.3390/biomimetics4020041)
Supplement: Supplementary file 1 [file biomimetics-04-00041-s001.pdf]

Supporting Information

Controlling the Antimicrobial Action of Surface Modified Magnesium Hydroxide Nanoparticles

Ahmed F. Halbus, Tommy S. Horozov, Vesselin N. Paunov

Contents

1. A schematic of the synthesis method of $Mg(OH)_2$NPs 2

2. The particles size and zeta potential of $Mg(OH)_2$NPs 3

3. Preparation and Characterization of $Mg(OH)_2$NPs 4

4. Fourier Transform Infrared Spectroscopy (FTIR) of $Mg(OH)_2$NPs 5

5. EDX diagram of *S. cerevisiae* cells with $Mg(OH)_2$NPs 6

6. EDX chart of the *C. reinhardtii* with $Mg(OH)_2$NPs 7

7. EDX chart of the *E. coli* with $Mg(OH)_2$NPs 9

8. Schematic representation of the bacterial cell wall 10

9. The antibacterial impact of $MgCl_2$ on *E. coli* 11

10. Comparison of the antimicrobial activity of uncoated and polyelectrolyte-coated $Mg(OH)_2$NPs on *S.cerevisiae*, *C. reinhardtii* and *E. coli* 12

11. Colony forming units (CFUs) of the bare $Mg(OH)_2$NPs and PAH-modified $Mg(OH)_2$NPs against cells 15

12. HEK 293 cell viability in the presence of bare $Mg(OH)_2$NPs and PSS/PAH-coated $Mg(OH)_2$NPs 19

13. Antimicrobial assay of SiO_2NPs on *E. coli* 20

14. Anti-yeast, anti-algal and antibacterial activity of free PAH in solution 21

1. **A schematic of the synthesis method of $Mg(OH)_2$NPs**

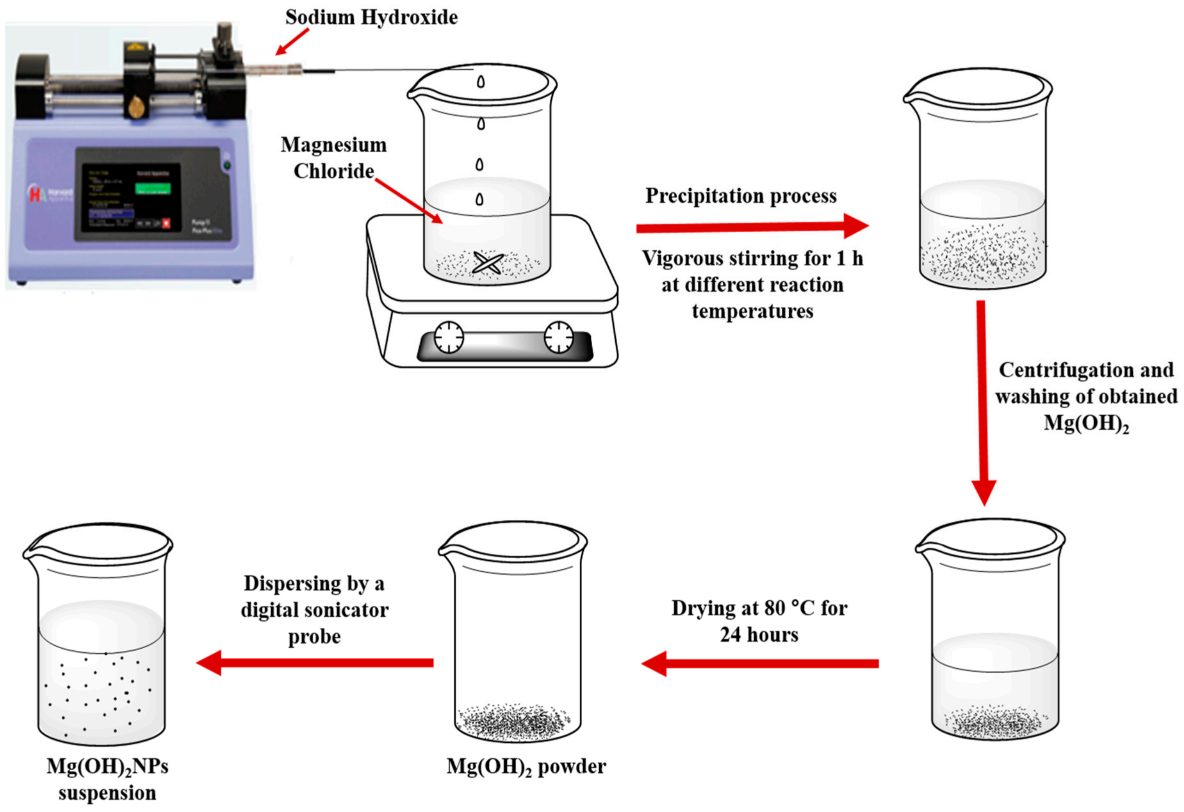

Figure S1. A schematic overview summarizing the synthesis method of $Mg(OH)_2$NPs.

2. **The particles size and zeta potential of $Mg(OH)_2$NPs**

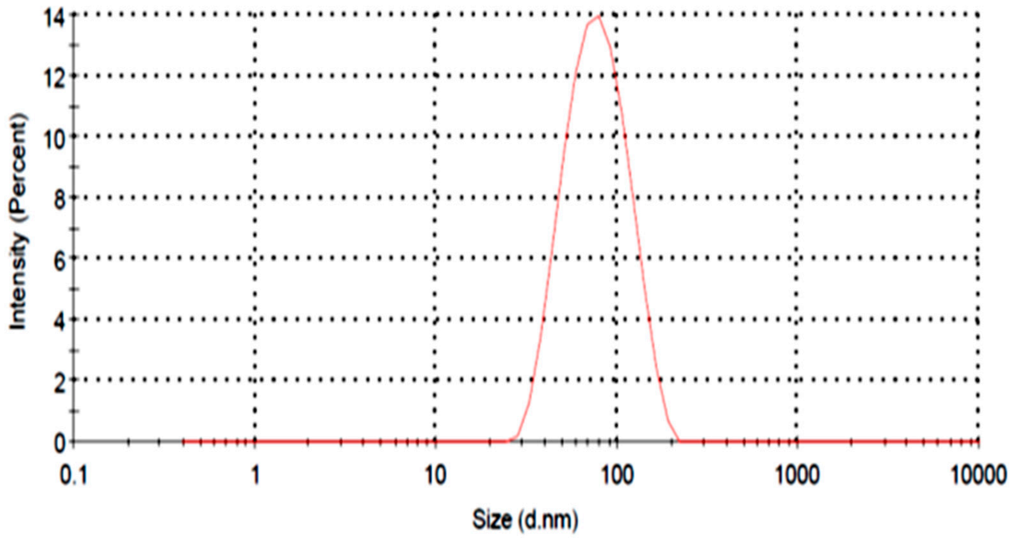

Figure S2. Particles size of $Mg(OH)_2$NPs made from a magnesium chloride at 75°C.

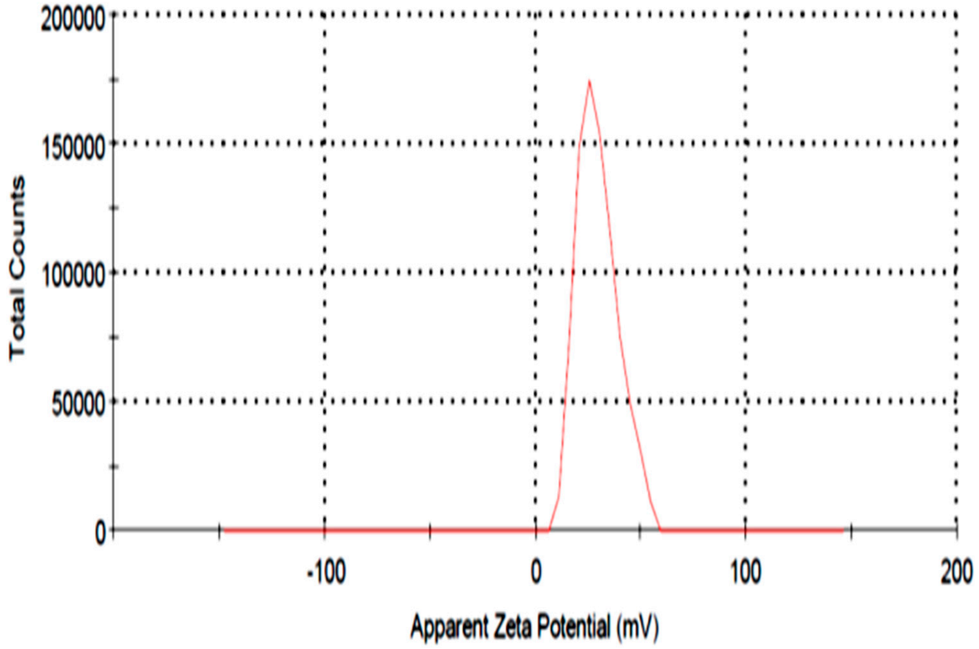

Figure S3. The zeta potential of $Mg(OH)_2$NPs made from a magnesium chloride at 75°C.

3. **Preparation and Characterization of $Mg(OH)_2$NPs**

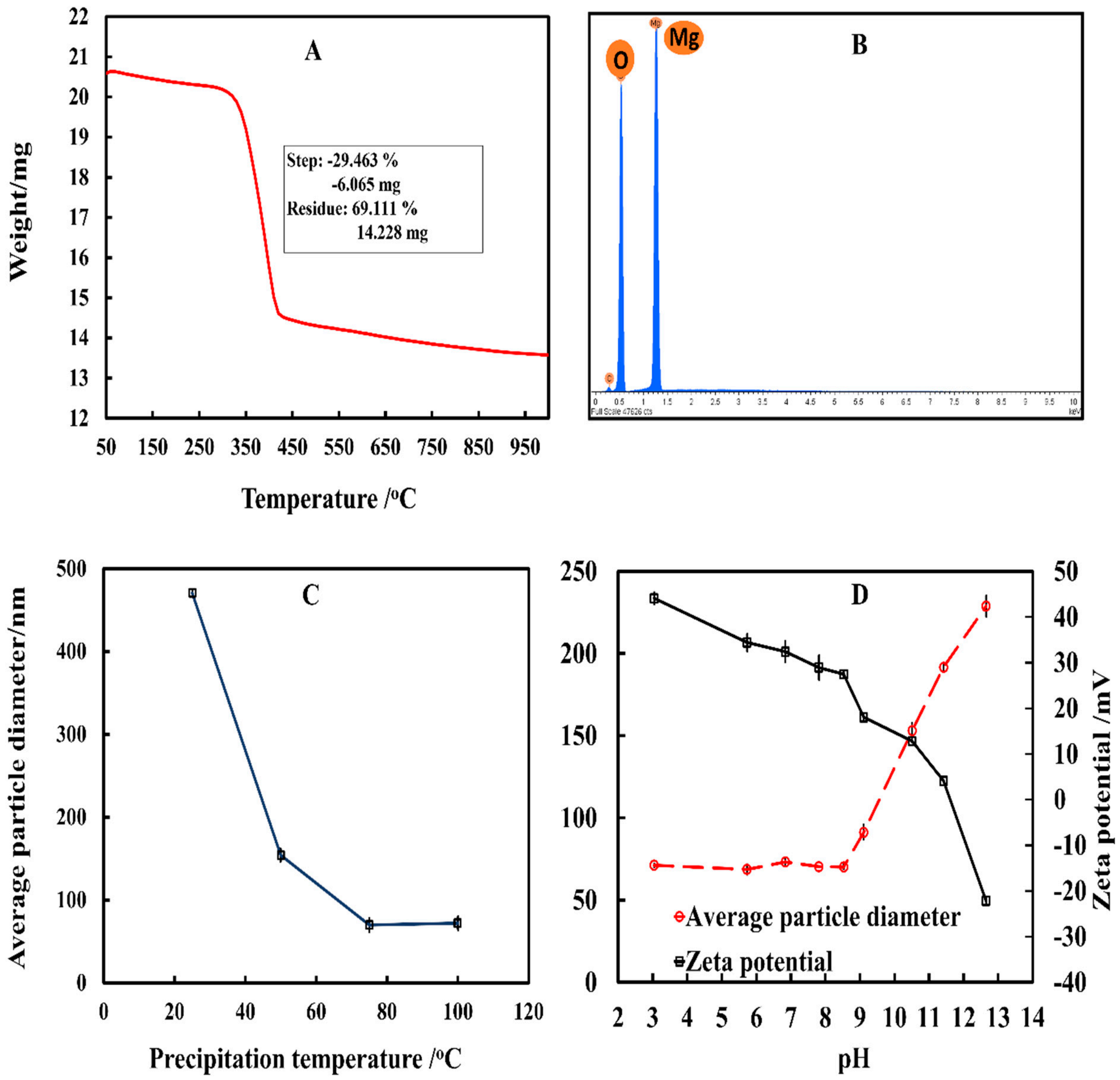

Figure S4. (A) Thermal gravimetric analysis pattern of $Mg(OH)_2$NPs powder. (B) The EDX spectra of the uncoated $Mg(OH)_2$NPs. (C) The impact of reaction temperature on the size of the produced $Mg(OH)_2$NPs. (D) Variations in particle size and zeta potential of $Mg(OH)_2$NPs suspensions with pH.

4. **Fourier Transform Infrared Spectroscopy (FTIR) of $Mg(OH)_2$NPs**

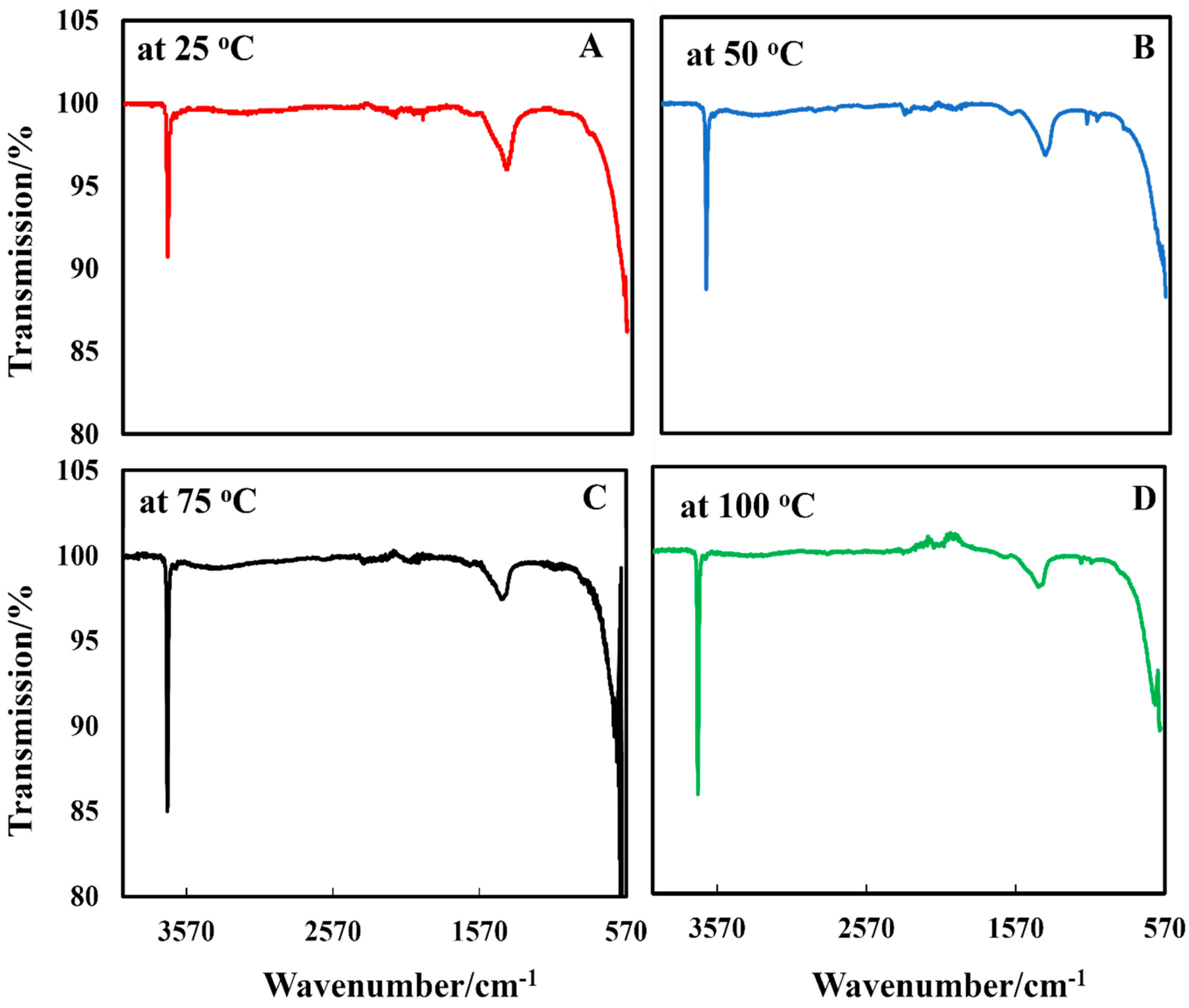

Figure S5. FTIR spectra of the as prepared $Mg(OH)_2$NPs at different reaction temperatures; (A) 25 ºC, (B) 50 ºC,(C) 75 ºC and (D) 100 ºC.

5. **EDX diagram of *S.cerevisiae* cells with $Mg(OH)_2$NPs**

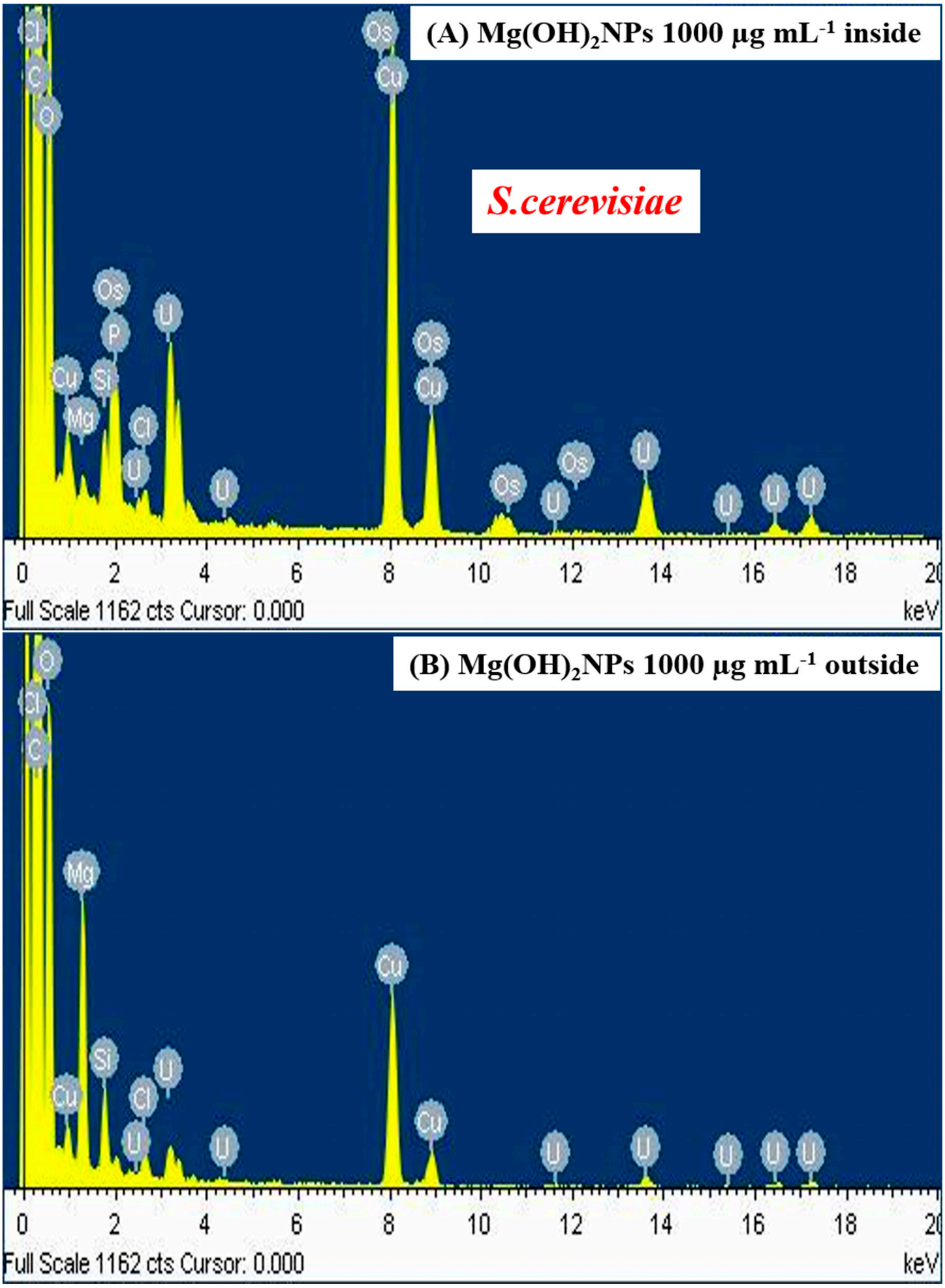

Figure S6. EDX diagram of *S.cerevisiae* cells at 1000 µg mL^{-1}: (A) the inside membrane of *S.cerevisiae* and (B) the outside membrane of *S.cerevisiae*. The data indicate the existence of $Mg(OH)_2$NPs on the outside part of the cell membrane.

6. **EDX chart of the *C. reinhardtii* with $Mg(OH)_2$NPs**

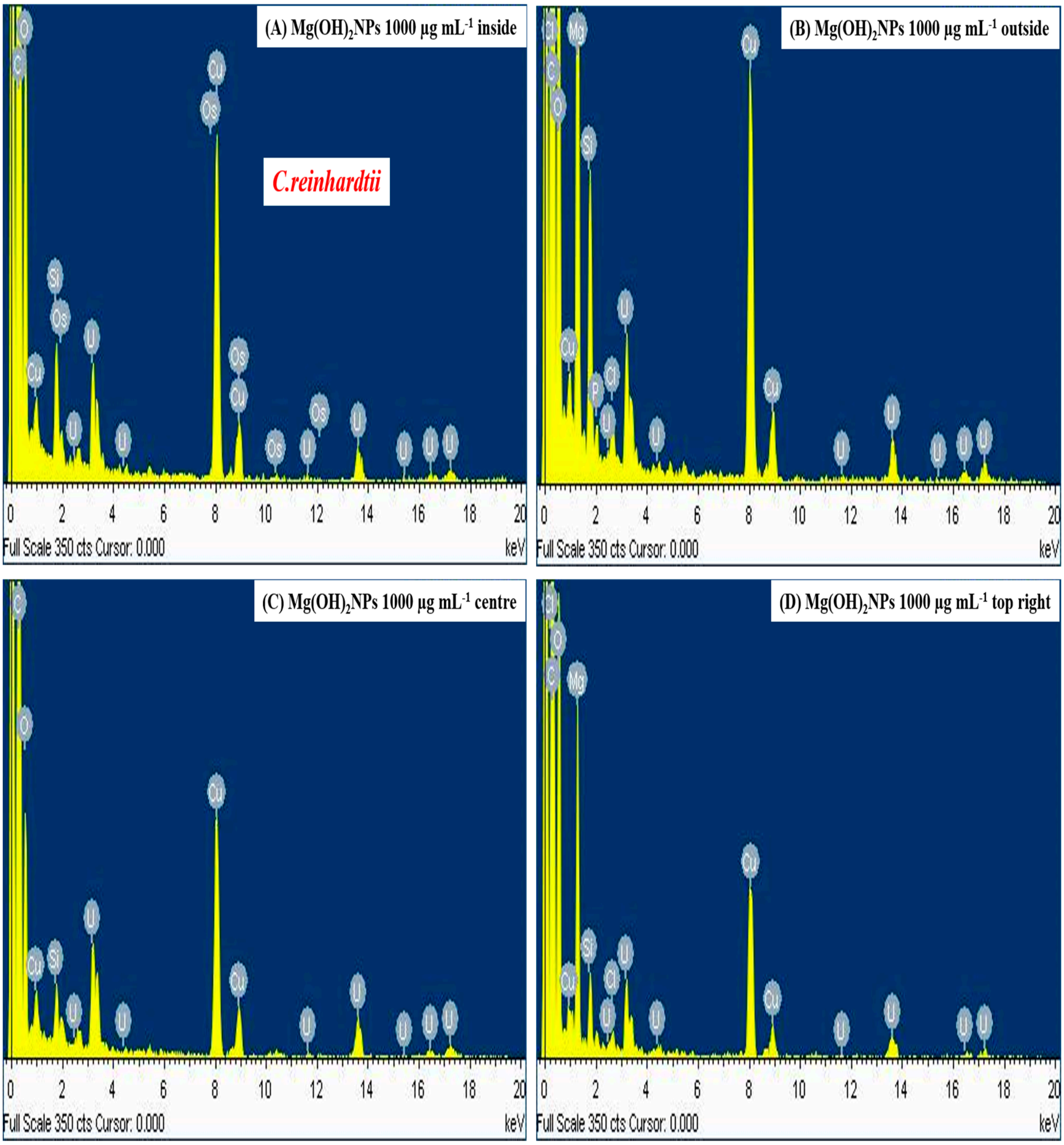

Figure S7. EDX chart of the *C. reinhardtii* with $Mg(OH)_2$NPs at 1000 µg mL^{-1}: (A) inside membrane of *C. reinhardtii* and (B) outside membrane of *C. reinhardtii* ; (C) centre cell and (D) top right. This demonstrates the lack of internalised $Mg(OH)_2$NPs in *C. reinhardtii* even at NPs concentration 1000 µg mL^{-1}.

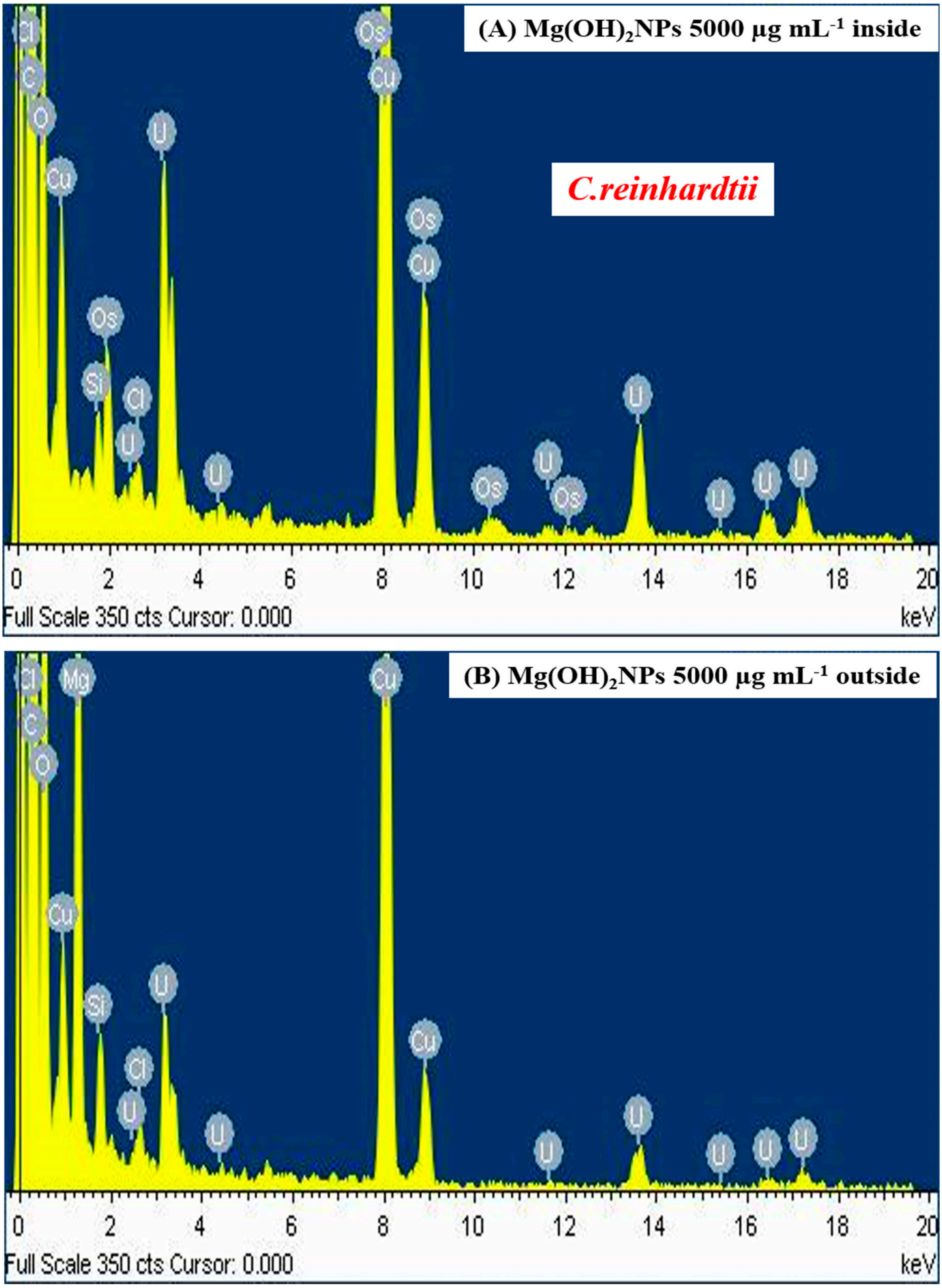

Figure S8. EDX chart of the *C. reinhardtii* with $Mg(OH)_2$NPs at 5000 µg mL^{-1}: (A) inside the membrane of the *C. reinhardtii* and (B) outer the membrane of the *C. reinhardtii*.

7. **EDX chart of the *E. coli* with $Mg(OH)_2$NPs**

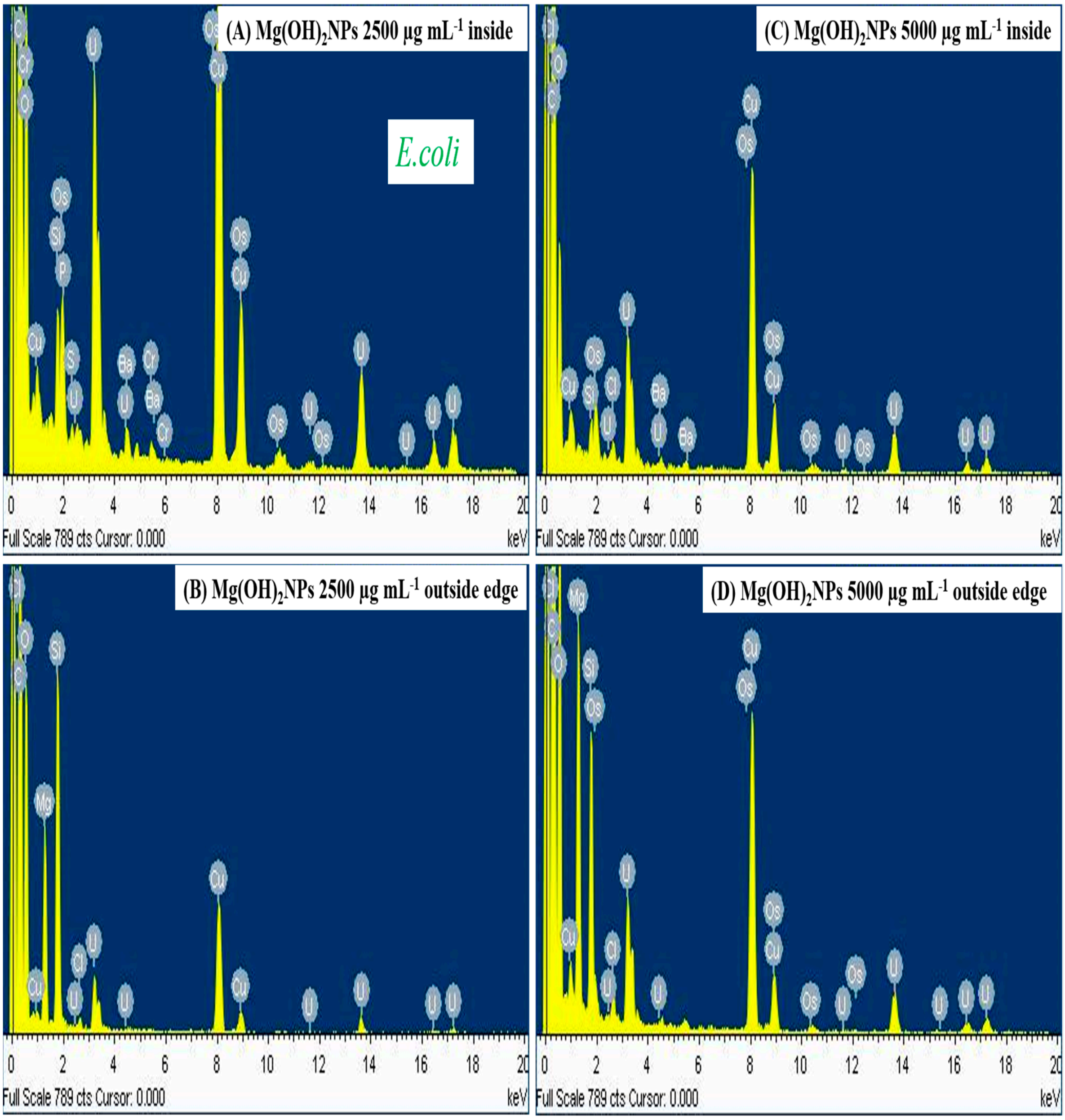

Figure S9. EDX diagram of *E. coli* cells incubated with $Mg(OH)_2$NPs at 2500 µg mL^{-1} and 5000 µg mL^{-1}: (A) *E. coli* inside wall and (B) *E. coli* outer wall areas. (C) *E. coli* inside wall and (D) *E. coli* outer wall areas. The data indicate the existence of $Mg(OH)_2$NPs on the external part of the cell membrane.

8. **Schematic representation of the bacterial cell wall**

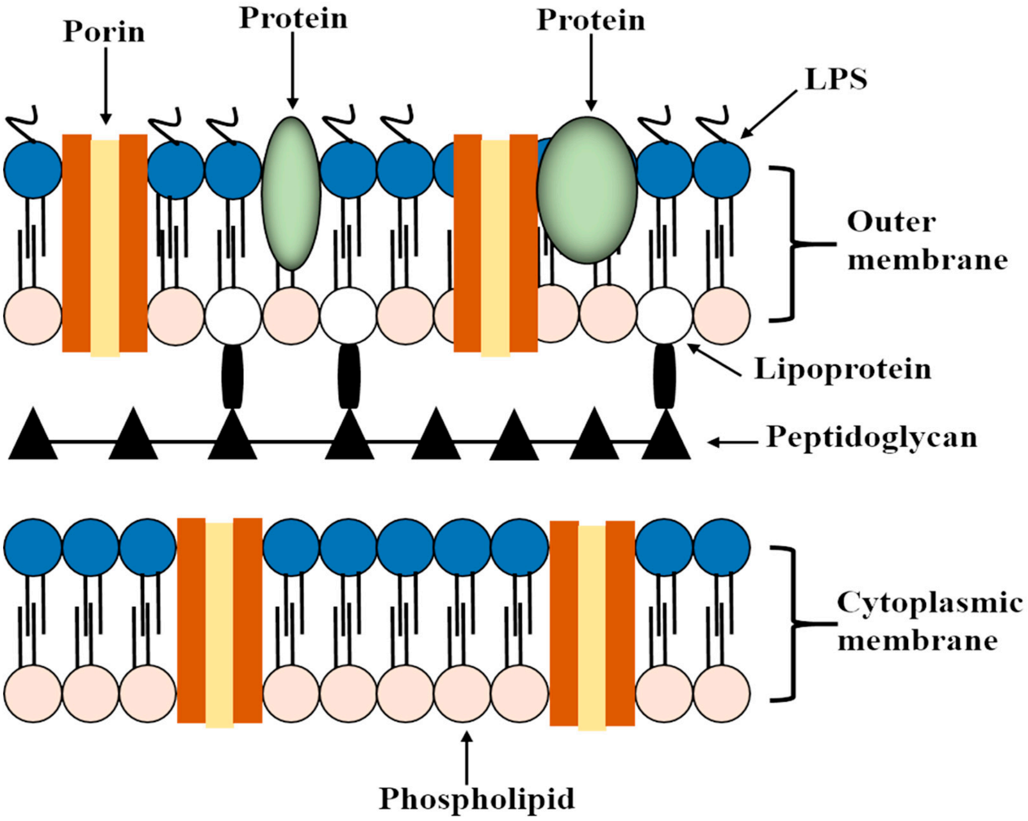

Figure S10. Schematic overview of the bacterial cell wall [37].

9. **The antibacterial impact of $MgCl_2$ on *E. coli***

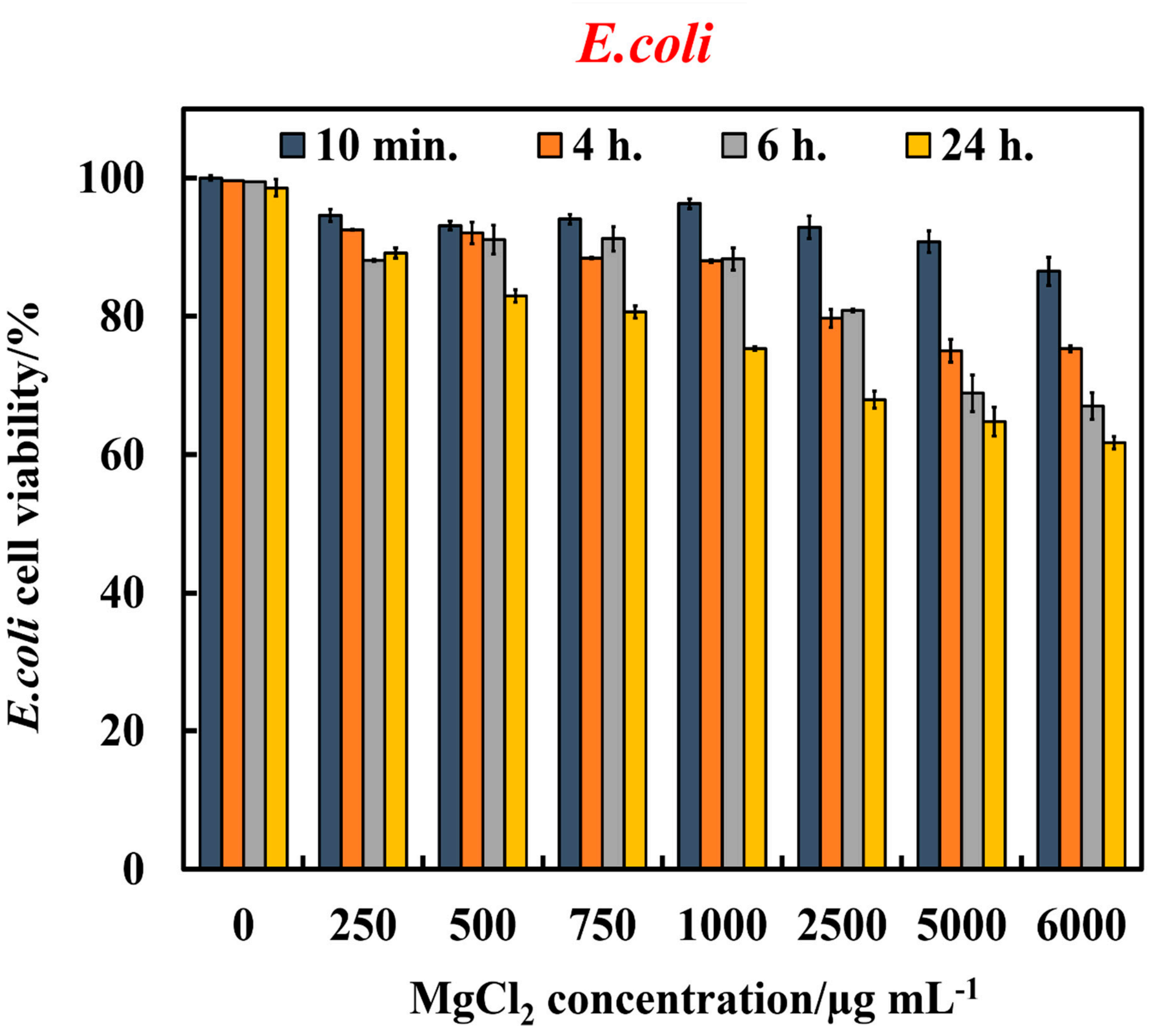

Figure S11. Antibacterial impact of various concentration of $MgCl_2$ towards *E. coli* for various exposure times. The experiment was achieved via incubated of *E. coli* with $MgCl_2$ for one day.

10. **Comparison of the antimicrobial activity of uncoated and polyelectrolyte-coated $Mg(OH)_2$NPs on *S.cerevisiae*, *C. reinhardtii* and *E. coli***

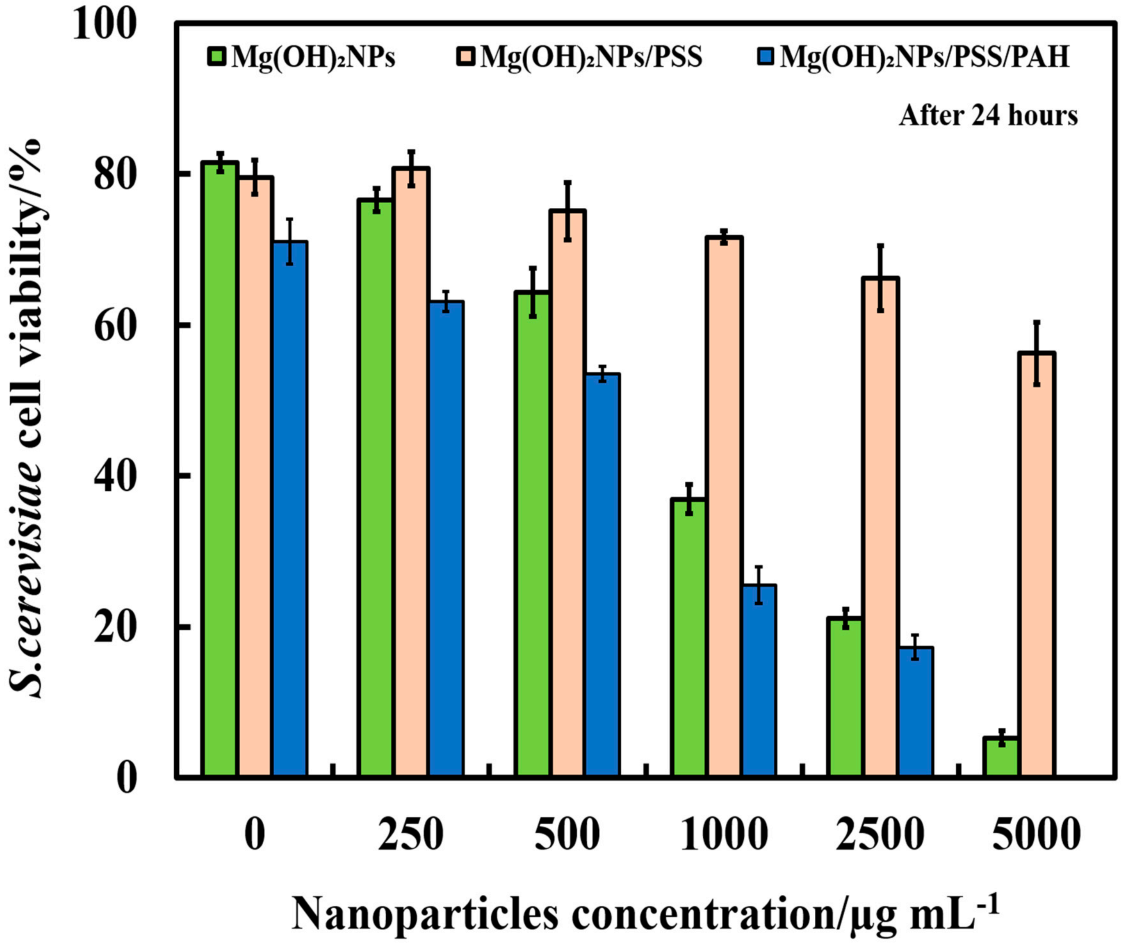

Figure S12. *S.cerevisiae* cell viability after incubation as a function of nanoparticle concentration for up to 24 hours with uncoated and polyelectrolyte-coated $Mg(OH)_2$NPs.

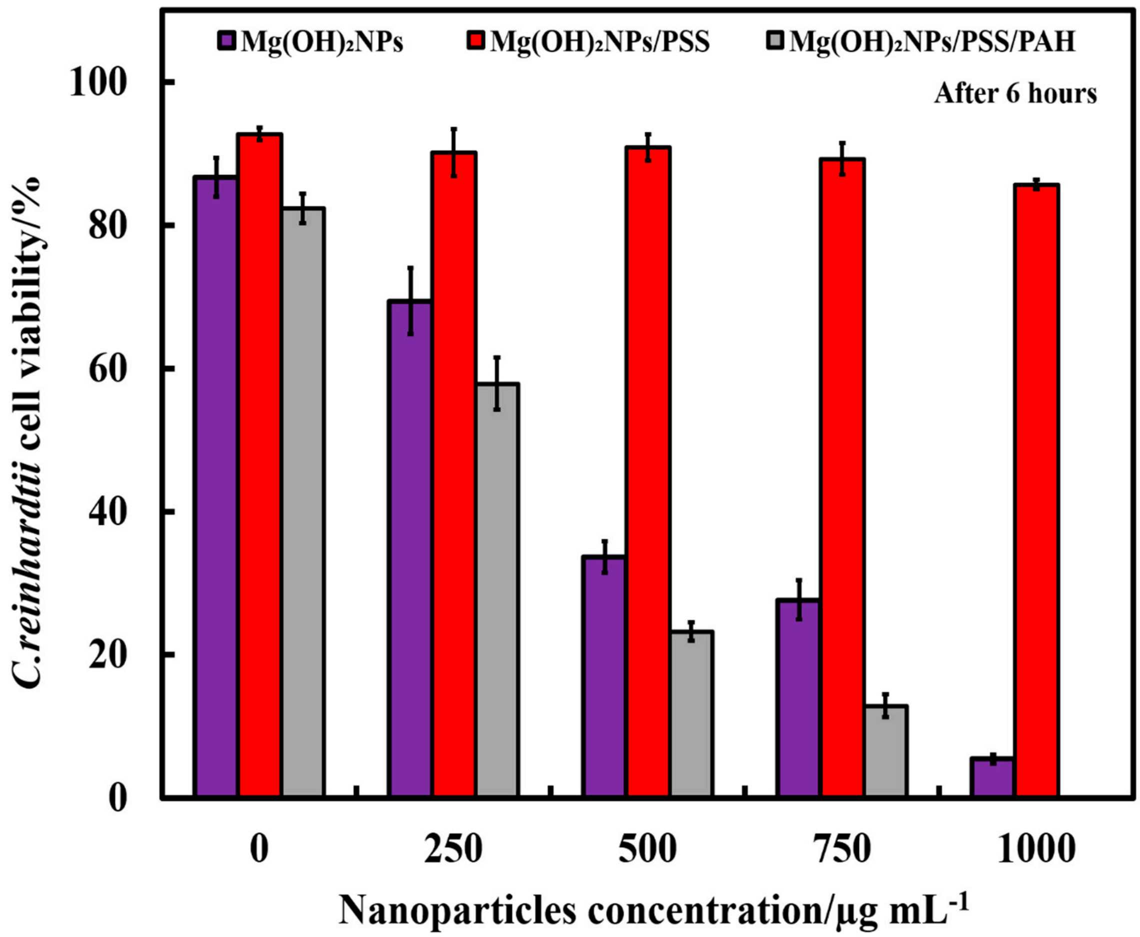

Figure S13. The antialgal activity of uncoated and polyelectrolyte-coated $Mg(OH)_2$NPs.

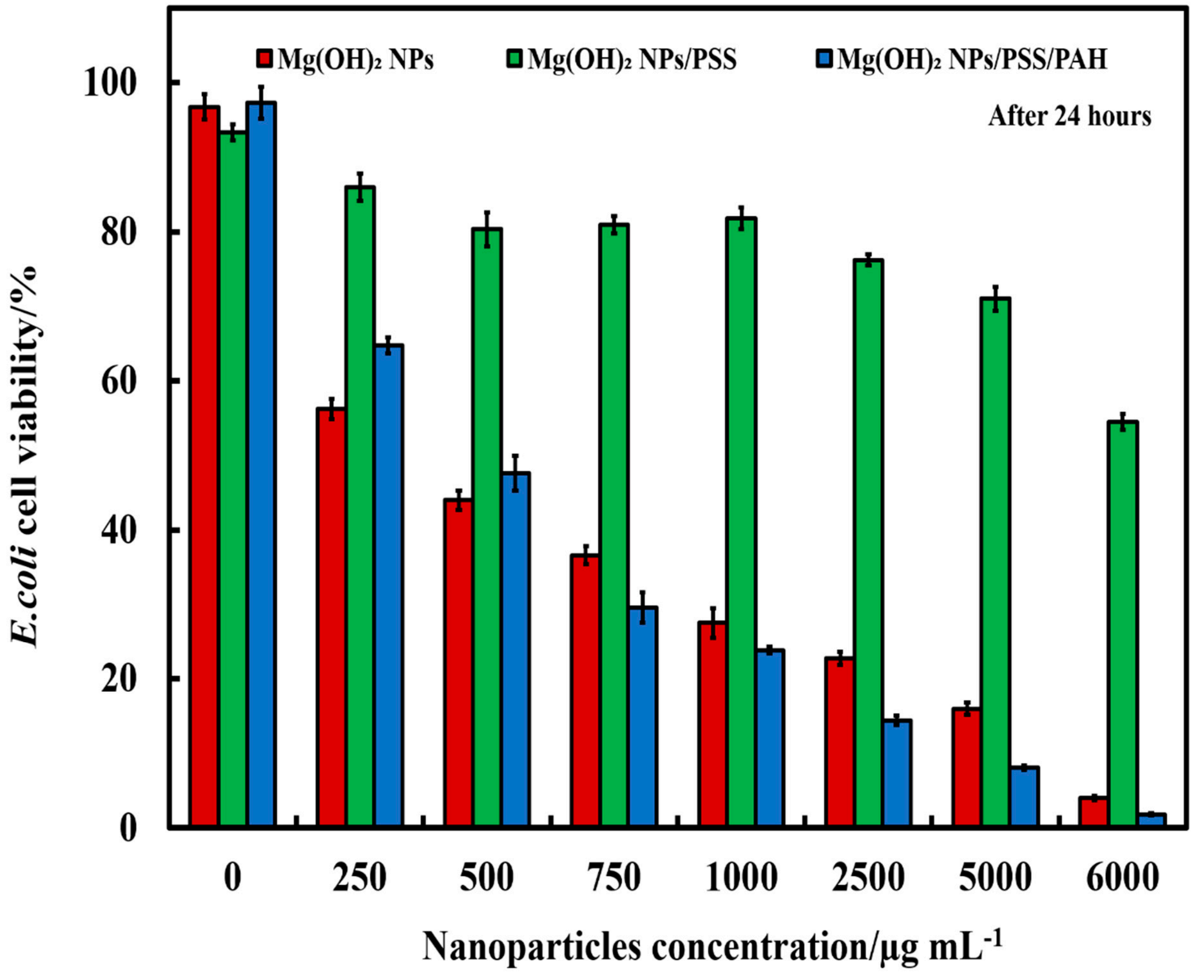

Figure S14. Relationship between the antibacterial efficiency of uncoated and polyelectrolyte-coated $Mg(OH)_2$NPs on the viability of *E. coli*. *E. coli* was incubated for one day to 0, 250, 500,750, 1000, 2500, 5000 and 6000 μg mL^{-1} of different types of nanoparticles.

11. Colony forming units (CFUs) of the bare $Mg(OH)_2$NPs and PAH-modified $Mg(OH)_2$NPs against cells

Figure S15, Figure S16, Figure S17 and Figure S18 shows the CFUs assay of *S. cerevisiae, C. reinhardtii* and *E. coli* where the control samples of untreated bacteria were compared with the ones treated with bare $Mg(OH)_2$NPs, $Mg(OH)_2$NPs/PSS and $Mg(OH)_2$NPs/PSS/PAH. The stock cultures of *C. reinhardtii* which were used for testing with typical concentration of 4×10^5 cells per mL and 9×10^5 cells per mL for *S. cerevisiae* determined by automatic cell counter (Cellometer Auto X4) and the *E. coli* bacterial culture stock was approximately 5×10^7 cells per mL.

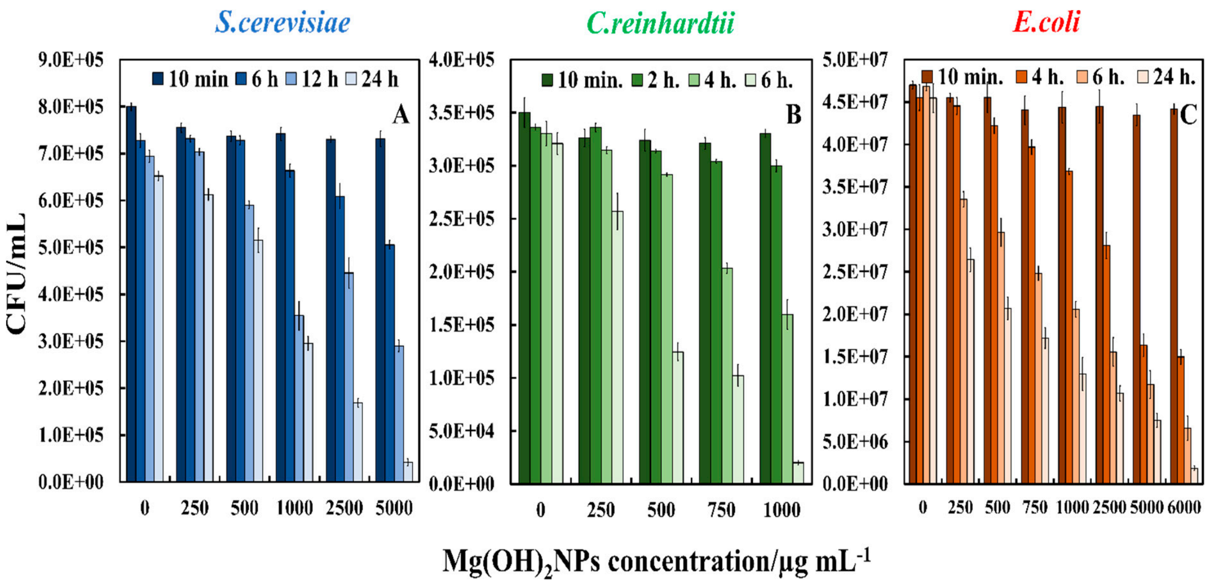

Figure S15. Colony forming unit (CFU) count of bare $Mg(OH)_2$NPs on (A) *S.cerevisiae* (B) *C. reinhardtii* and (C) *E. coli* at various particle concentrations. The cells were incubated with the $Mg(OH)_2$NPs at different periods of time shown.

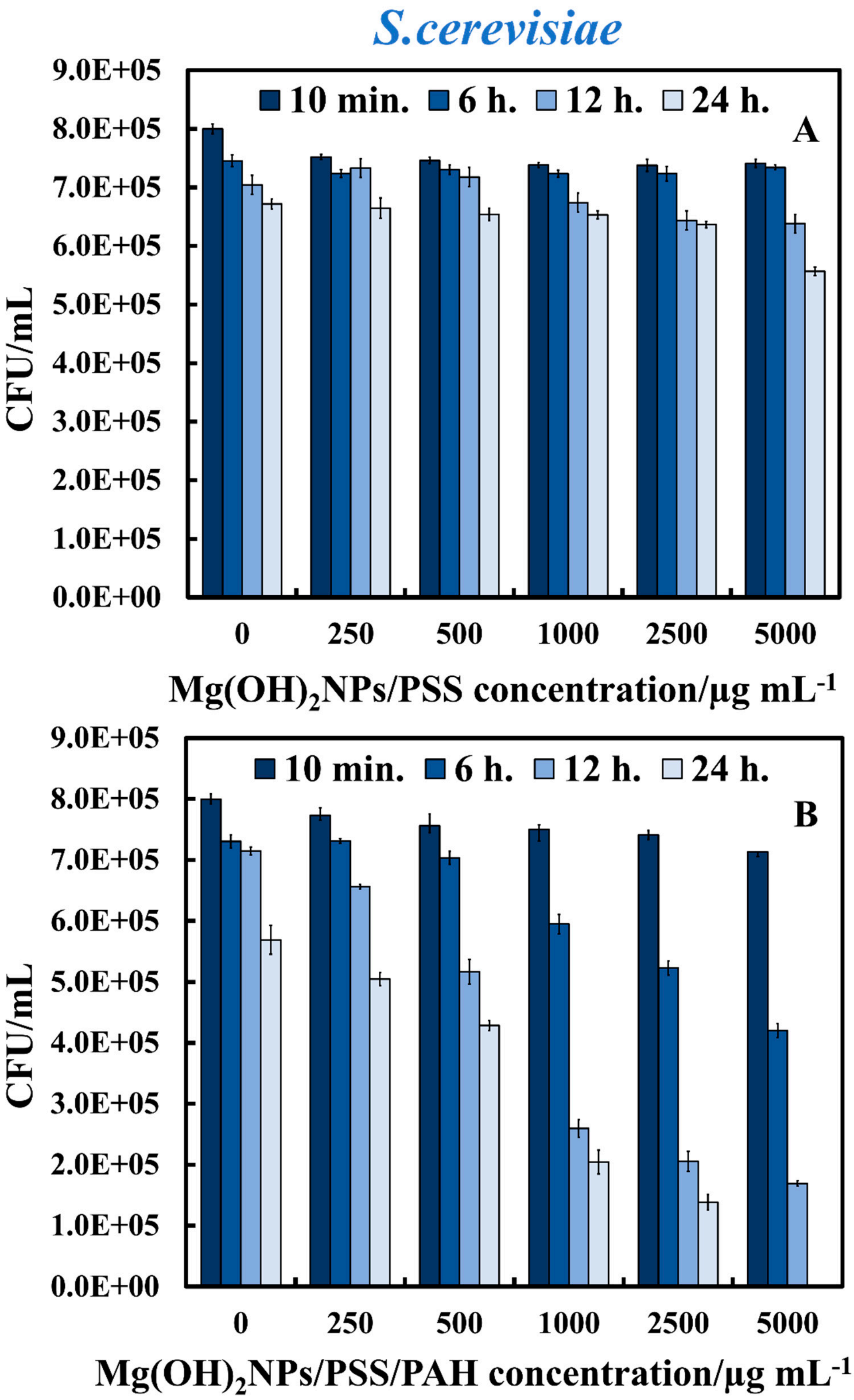

Figure S16. Colony forming unit (CFU) count of *S.cerevisiae* as a function of nanoparticle concentration after incubation for up to 24 hours with (A) $Mg(OH)_2$NPs/PSS and (B) $Mg(OH)_2$NPs/PSS/PAH.

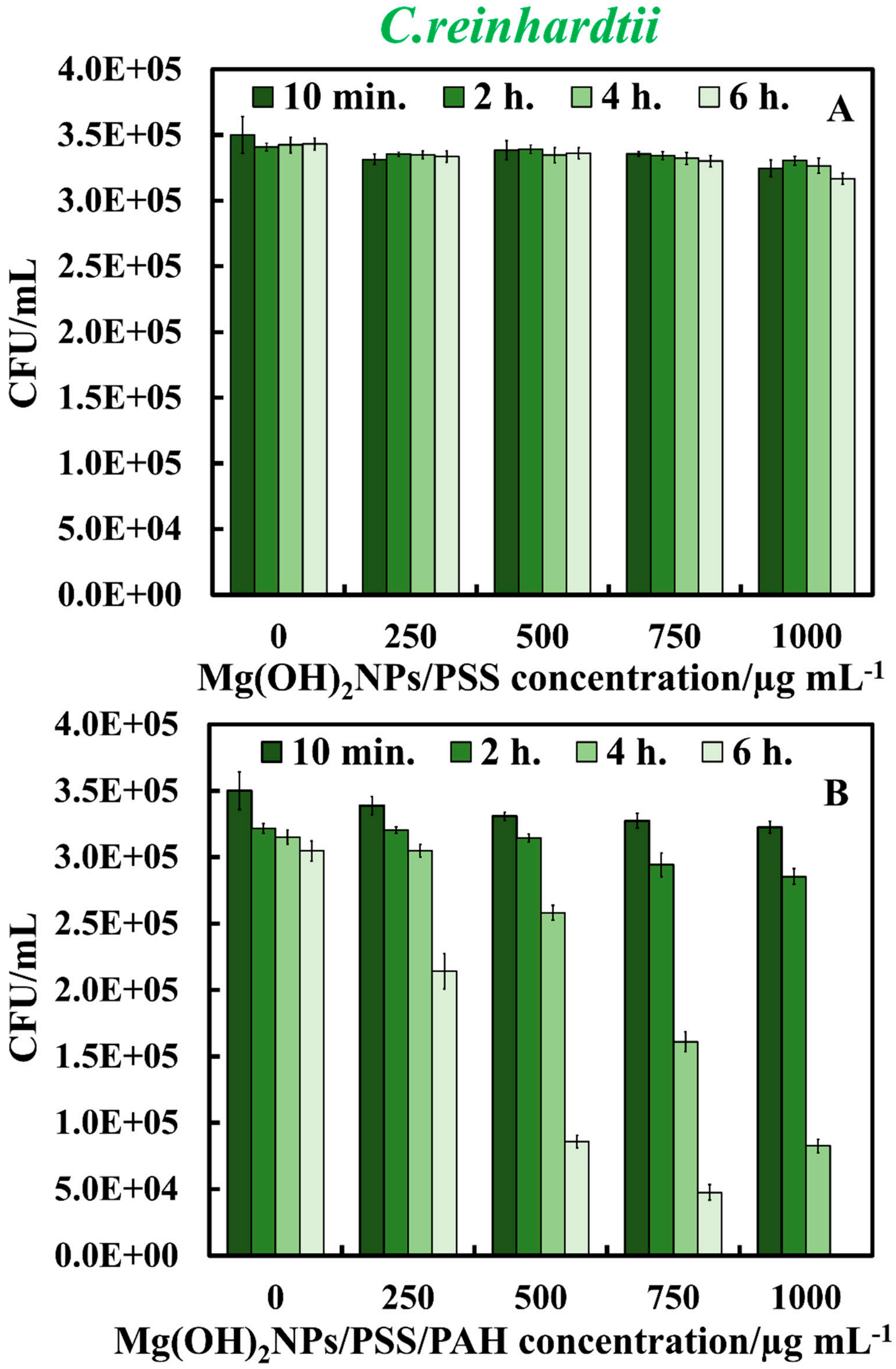

Figure S17. Colony forming unit (CFU) count of *C. reinhardtii* as a function of nanoparticle concentration after incubation for up to 6 h with (A) $Mg(OH)_2$NPs/PSS and (B) $Mg(OH)_2$NPs/PSS/PAH.

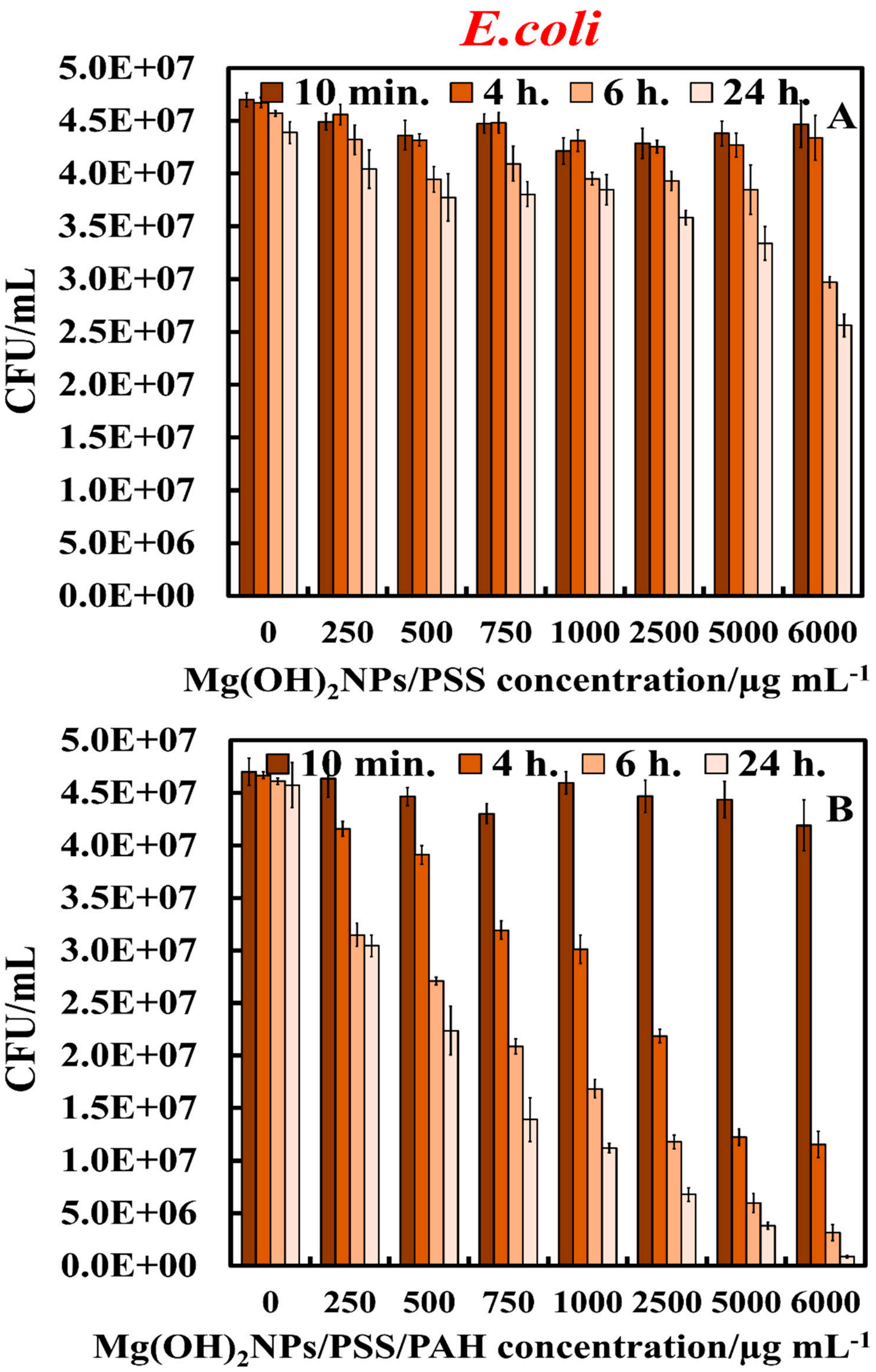

Figure S18. Colony forming unit (CFU) count of *E. coli* after treatment with (A) $Mg(OH)_2$NPs/PSS and (B) $Mg(OH)_2$NPs/PSS/PAH for various incubation times as a function of the NPs concentration.

12. HEK 293 cell viability in the presence of bare $Mg(OH)_2$NPs and PSS/PAH-coated $Mg(OH)_2$NPs

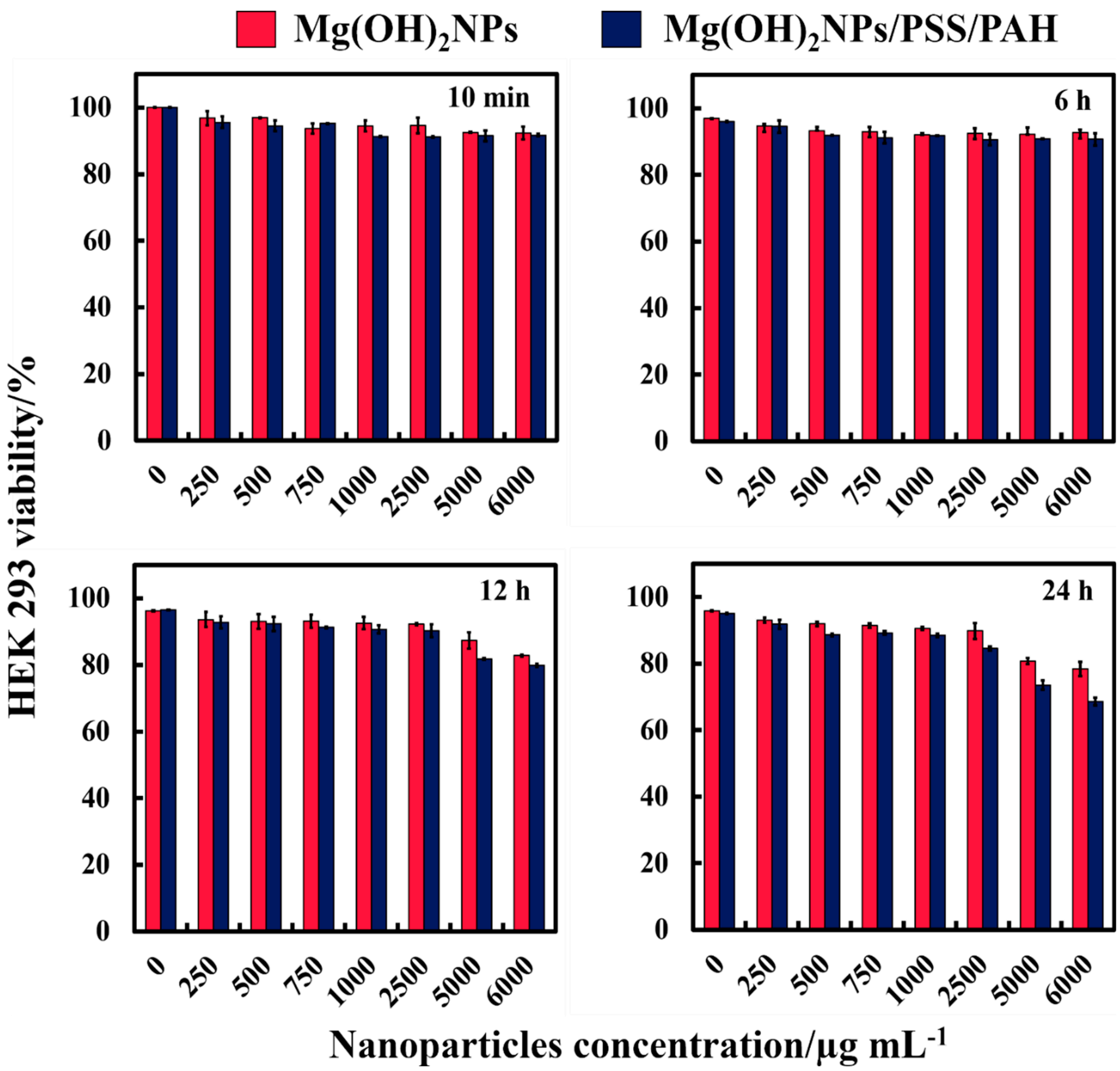

Figure S19. Comparison of the cell viability of human embryonic kidney cells (HEK 293 cell line) upon incubation as a function of nanoparticle concentration for up to 24 h with bare $Mg(OH)_2$NPs and $Mg(OH)_2$NPs/PSS/PAH.

13. Antimicrobial assay of SiO_2NPs on *E. coli*

Figure S20 shows the antimicrobial assay of SiO_2NPs on *E. coli* for up to 24 hours of exposure at different SiO_2NPs concentrations. We used SiO_2NPs as a negative control for comparing the toxicity of the $Mg(OH)_2$NPs. One can see a very small effect on the presence of bare SiO_2NPs on the *E. coli* viability. Note that even the control sample of *E. coli* have lost some of their viability over this period of time due to depletion of the media.

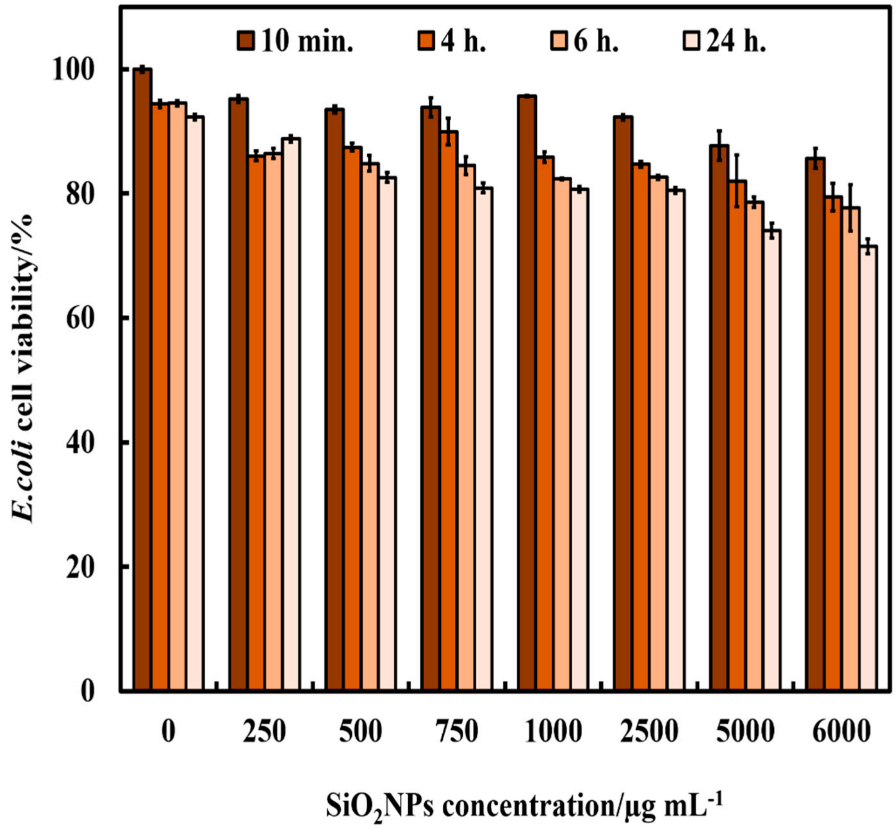

Figure S20. Antibacterial activity of SiO_2NPs at various concentrations on *E. coli*. The *E. coli* cells were incubated with the SiO_2NPs at 10 min, 4 h, 6 h and 24 h of exposure before being washed and tested for their cell viability.

14. Anti-yeast, anti-algal and antibacterial activity of free PAH in solution

There is no free PAH in the PSS/PAH-coated $Mg(OH)_2$NPs suspension since we wash the PSS/PAH-coated $Mg(OH)_2$NPs multiple times by centrifugation and discard the supernatant. To check possible effects from leaching polyelectrolytes we did antimicrobial testing of free PAH on *S. cerevisiae*, *C. reinhardtii* and *E. coli.* Figure S21 shows the antimicrobial assay of the free PAH on *S. cerevisiae*, *C. reinhardtii* and *E. coli* for up to 6 hours for *C. reinhardtii* and 24 hours for *S. cerevisiae* and *E. coli* of exposure. Both runs were done at the varying overall PAH concentration and different incubation times. One can see a very small effect on the presence of free PAH on the *cells* viability. One can conclude that the free PAH does not measurably impact the cell viability up to 5000 μg mL^{-1} for *S. cerevisiae,* 1000 μg mL^{-1} for *C. reinhardtii* and 6000 μg mL^{-1} for *E. coli*. Note that in our $Mg(OH)_2$NPs/PSS/PAH nanoparticles there is not ant free PAH and free PSS as the particles have undergo multiple washing/centrifugation cycles after their surface functionalization. However, at these concentrations of the PAH- coated on $Mg(OH)_2$NPs, the effect of the $Mg(OH)_2$NPs on *S. cerevisiae*, *C. reinhardtii* and *E. coli* is very significant – see Figure 4, Figure 9, Figure 10 and Figure 11 in the main paper, respectively. Therefore, one may conclude that the PSS/PAH-coated $Mg(OH)_2$NPs show excellent anti-yeast, anti-algal and antibacterial with these cells which is not related to free PAH.

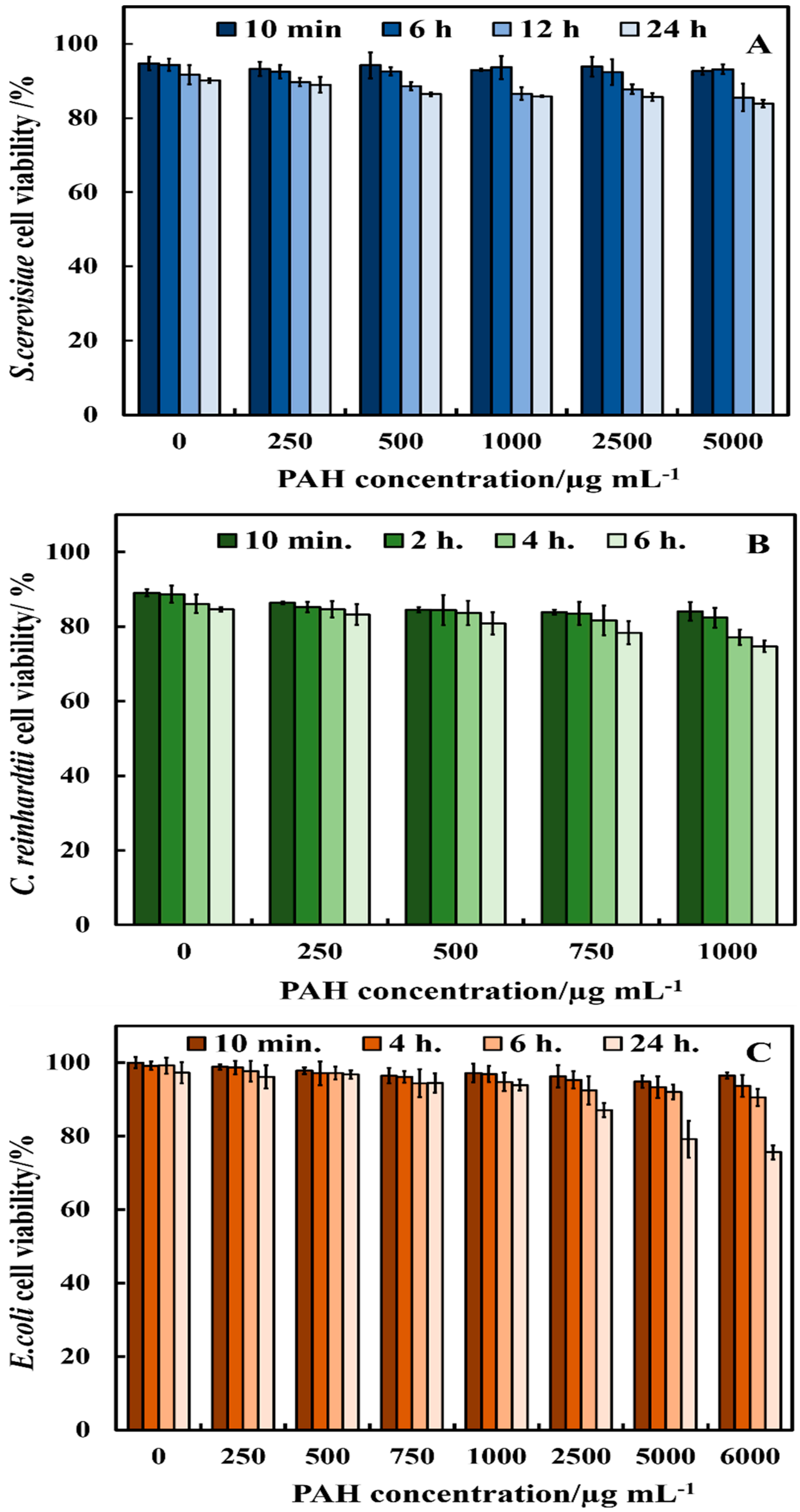

Figure S21. The anti-yeast, anti-algal and antibacterial activity of free PAH at various concentrations on (A) *S. cerevisiae,* (B) *C. reinhardtii* (C) *E. coli*. The cells were incubated with the free PAH at different times of exposure before being washed and tested for their cell viability.
